# Highly Conductive Ink Based on Self‐Aligned Single‐Walled Carbon Nanotubes through Inter‐Fiber Sliding in Cellulose Fibril Networks

**DOI:** 10.1002/advs.202402854

**Published:** 2024-08-28

**Authors:** Sejung Park, Yeeun Song, Boeun Ryu, Young‐Woong Song, Haney Lee, Yejin Kim, Jinsub Lim, Doojin Lee, Hyeonseok Yoon, Changkee Lee, Changhun Yun

**Affiliations:** ^1^ School of Polymer Science and Engineering Chonnam National University Gwangju 61186 Republic of Korea; ^2^ Department of Materials Science and Engineering Chonnam National University Gwangju 61186 Republic of Korea; ^3^ Korea Institute of Industrial Technology (KITECH) Gwangju 61012 Republic of Korea; ^4^ Korea Institute of Industrial Technology (KITECH) Ansan‐si 15588 Republic of Korea

**Keywords:** cellulose, conductive nanocomposite, self‐alignment, single‐walled carbon nanotube, structural rearrangement

## Abstract

Carbon nanotubes (CNTs), owing to their superior electrical and mechanical properties, are a promising alternative to nonmetallic electrically conducting materials. In practice, cellulose as a low‐cost sustainable matrix has been used to prepare the aqueous dispersion of cellulose‐CNT (C‐CNT) nanocomposites. However, the compatibility with conventional solution‐processing and structural rearrangement for improving conductivity has yet to be determined. Herein, a straightforward route to prepare a conductive composite material from single‐walled CNTs (SWCNTs) and natural pulp is reported. High‐power shaking realizes the self‐alignment of individual SWCNTs in a cellulose matrix, resulting from the structural change in molecular orientations owing to countless collisions of zirconia beads in the aqueous mixture. The structural analysis of the dried C‐CNT films confirms that the entanglement and dispersion of C‐CNT nanowires determine the mechanical and electrical properties. Moreover, the rheological behavior of C‐CNT inks explains their coating and printing characteristics. By controlling shaking time, the electrical conductivity of the C‐CNT films with only 9 wt.% of SWCNTs from 0.9 to 102.4 S cm^−1^ are adjusted. the optimized C‐CNT ink is highly compatible with the conventional coating and printing processes on diverse substrates, thus finding potential applications in eco‐friendly, highly flexible, and stretchable electrodes is also demonstrated.

## Graphical abstract

This paper presents a novel and straightforward route to prepare an eco‐friendly nonmetallic conducting ink using natural cellulose and SWCNTs. The self‐alignment of individual SWCNTs in a cellulose matrix, resulting from the structural change in molecular orientations, realizes a highly conductive nanocomposite. The prepared nanocomposite ink is highly compatible with the conventional coating and printing processes on diverse substrates.

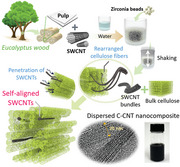

## 1 Introduction

The requirements for nonmetallic electrical conductors have been increasing in various applications, such as optoelectronics, wearable electronics, energy‐storage devices, and bioelectronics.^[^ ^1^ ^]^ Among the potential alternatives to metallic compounds, conductive layers prepared using a family of metal oxides, conducting polymers, and carbons have attracted considerable interest owing to their electrochemical stability without inevitable chemical reactions, reduced environmental contamination, and sustainability in material usage.^[^ ^2^ ^]^ However, challenges remain owing to poor mechanical stability and electrical conductivity, unlike in the case of conventional metallic conductors.^[^ ^2a,c^ ^]^ Carbon‐based nanomaterials such as fullerenes, carbon nanotubes, and graphene are suitable candidates for replacing conductive agents because these nanomaterials have better electrical and mechanical properties than metal oxides and conducting polymers.^[^ ^1^, ^3^ ^]^ Especially, individual carbon nanotubes (CNTs) are among the most electrically conductive materials.^[^ ^3^, ^4^ ^]^ For example, the electrical conductivities of single‐walled CNTs (SWCNTs) and multi‐walled CNTs (MWCNTs) have been reported to be of the order of 10^2^–10^6^ and 10^3^–10^5^ S cm^−1^, respectively.^[^ ^3^, ^4^ ^]^ However, the electrical conductivities of bulk CNT materials prepared using the conventional manufacturing process are considerably lower than that of an individual CNT.^[^ ^4^ ^]^ Several researchers have applied individual or bundles of CNTs as reinforcing components for improving or tailoring the electrical properties of nanocomposites.^[^ ^5^ ^]^ Although several studies related to CNT/polymer nanocomposites have been conducted, studies on the uniform and high‐quality dispersion of individual CNTs in polymer matrices for realizing metal‐free electrical conductors remain limited.^[^ ^3^, ^6^ ^]^ Among the reported CNT/polymer combinations with thermoplastic polymers (such as polystyrene, polyvinylidene difluoride, polyvinylpyrrolidone, and biaxially oriented polypropylene) and thermoset polymers (such as epoxy resin, and polyimide), a conductive nanocomposite consisting of low‐cost and biodegradable cellulose nanofibers (CNFs) as a polymer matrix has been extensively studied.^[^ ^1^, ^2^, ^3^, ^7^, ^8^ ^]^ The nanostructured CNF‐CNT composite was generally prepared after dispersing the nanocomposites in an aqueous solution by chemically functionalizing CNT or CNF or using a chemical additive or a surfactant.^[^ ^4^, ^5^, ^9^ ^]^ However, such a chemical treatment in the mixture solution substantially reduced the electrical properties of the CNF‐CNT nanocomposites.^[^ ^3^, ^10^ ^]^ Instead, Mahiar et al. demonstrated the concept of CNF‐assisted aqueous dispersions of SWCNTs for the first time; they demonstrated that CNFs are an excellent dispersion agent for SWCNTs in aqueous solutions.^[^ ^3a^ ^]^ Moreover, a stable aqueous colloidal dispersion with up to 75 wt.% of SWCNTs was prepared via the electrostatically stabilized association between CNFs and CNTs in water, and the resulting dried nanocomposite had an electrical conductivity of 515 S cm^−1^.^[^ ^3c^ ^]^ However, the compatibility of conductive ink containing a colloidal dispersion of CNF‐CNT with conventional solution‐processing technologies such as printing, dispensing, and coating has not been determined thus far.^[^ ^3^, ^11^ ^]^ In addition, the structure of assembled SWCNTs in a polymer matrix based on cellulose fibrils has barely been reported. However, the nanostructure of a conducting reinforcement can affect the electrical characteristics of a CNT‐based composite material.^[^ ^3^, ^9^ ^]^

This study presents a simple and straightforward route for preparing a non‐metallic conductive ink containing a composite material consisting of SWCNTs and natural cellulose fibrils. For the first time, we identified that the mechanical energy transferred from the collision of zirconia beads in an aqueous mixture resulted in the formation of a SWCNT‐nanocomposite consisting of a polymer matrix of natural cellulose from Eucalyptus pulp without requiring any chemical treatment or additives. Structural analysis indicated that individual SWCNTs penetrated the cellulose fibril network and aligned along the chain direction of the cellulose fibrils during the collision process. Additionally, consecutive changes in the nanostructure depended on the processing time of the collision. Furthermore, the rheological analysis revealed that the prepared aqueous ink was highly compatible with conventional solution processing and demonstrated the consistency of the generated nanostructures with the SWCNT‐reinforced cellulose fibers in water. The optimization of the ink‐preparation process yielded a nanocomposite film with a high electrical conductivity of 102 S cm^−1^ (362 S cm^−1^) by using only 9 wt.% (33 wt.%) of SWCNTs, which can be fabricated by the coating and dispensing processes. In addition, we investigated the performance of the SWCNT‐cellulose composite in various applications, including a stretchable conducting wire for electrical devices and a current‐collecting layer for all‐solid‐state batteries.

## 2 Results and Discussion

Several synthetic polymer matrices have been studied for preparing nonmetallic composite materials using nanostructured carbon.^[^ ^1^, ^2^, ^7^ ^]^ However, to avoid chemical additives and expensive surface‐treatment processes of reinforced carbon nanomaterials, many researchers selected cellulose as the matrix polymer and dispersion agent for SWCNTs or MWCNTs.^[^ ^3^, ^8^, ^9^, ^11^, ^12^ ^]^ Cellulose is the most abundant polymer in nature and has been used extensively as a sustainable building block for functional and structural materials like paper.^[^ ^13^, ^14^ ^]^ Additionally, cellulose has many attractive features such as nontoxicity, renewability, biodegradability, low cost, and colloidal stability.^[^ ^14^ ^]^ Moreover, cellulose can be used in various electronic applications because of its unique dielectric properties and excellent biocompatibility.^[^ ^13^, ^15^ ^]^ In particular, electrically conducting composites based on CNFs and CNTs show excellent mechanical and electrical characteristics due to the complementary mechanical properties of the cellulose nanomaterial.^[^ ^1^, ^8^, ^11^ ^]^ CNFs can be prepared by fractionating cellulose resources such as plants, wood, algae, tunicate, and bacteria with polysaccharide structures, which comprise intrinsically hierarchical structures from nanocelluloses. CNFs generally require expensive chemical (i.e., oxidation, acid hydrolysis, and electro‐spinning), mechanical (i.e., sonication and cryo‐crushing), and enzymatic (i.e., endonucleases and exonucleases) pre‐treatment processes.^[^ ^15^, ^16^ ^]^ However, such pre‐treatment processes always introduce additional environmental pollutants and cause substantial losses in manufacturing energy.^[^ ^15^, ^16^ ^]^

**Figure 1:**
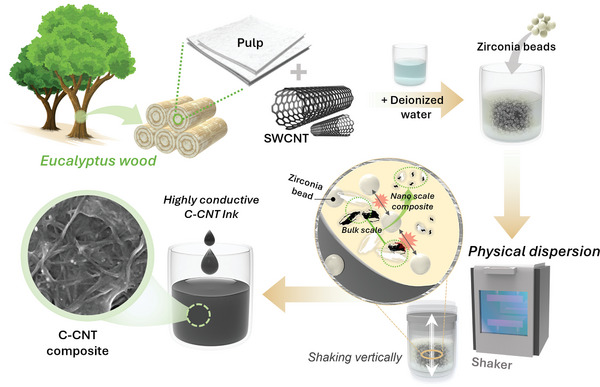
Schematics of the preparation process of the highly conductive aqueous ink (C‐CNT) consisting of cellulose and single‐walled carbon nanotubes without any chemical additives or surface functionalization. The C‐CNT ink can be made from natural wood pulp and a powder of single‐walled carbon nanotube in de‐ionized water using the mechanical shaking process with zirconia beads. The resultant C‐CNT ink prepared using an eco‐friendly, low‐cost method showed high‐quality dispersions of C‐CNT nanocomposites in water. Details regarding the preparation can be found in the experimental section.

**Figure 2:**
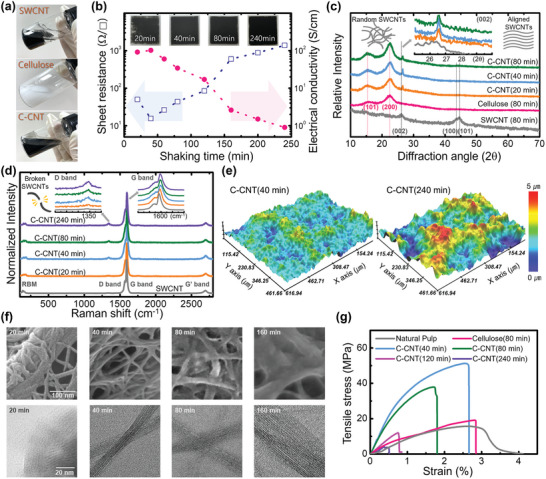
a) SWCNTs, pulp, and the prepared C‐CNT composite in water after shaking for 40 min. b) Change in the measured sheet resistance (rectangular) and corresponding electrical conductivity (circle) depending on the shaking time. The insets are tested C‐CNT films prepared by drop casting 0.3 ml of C‐CNT ink on a glass substrate. c) X‐ray diffraction (XRD) patterns of the SWCNTs, the cellulose film, and the C‐CNT films obtained under various shaking times. The inset graphs are enlarged XRD patterns of the C‐CNTs showing the (002) peak corresponding to the SWCNTs. d) Raman spectra of the SWCNTs and the C‐CNT films under various shaking times. The inset graphs show the enlarged spectra of the D‐band (left) and G‐band (right) from the SWCNTs. e) 3D surface‐morphological images of the C‐CNT films obtained using two different shaking times (40 and 240 min) using a 3D optical surface profiler. f) High‐resolution SEM (top) and TEM images (bottom) of the C‐CNT films obtained under various shaking times. g) Tensile stress‐strain curves of the natural pulp, a cellulose film obtained using a shaking time of 80 min, and the C‐CNT films obtained using various shaking times.

We proposed a novel preparation method to prepare a nanocomposite with a high electrical conductivity using natural cellulose and SWCNTs, as depicted in **Figure** **1**. Unlike in previous studies, we focused on forming an aqueous ink by dispersing the nonmetallic conducting material in water because eco‐friendly conductive inks have garnered considerable interest in industry and academia.^[^ ^1,a,c,d,g^ ^]^ The most significant advantage of the proposed process shown in Figure 1 is that we started from the bulk cellulose material derived from Eucalyptus pulp instead of the most commonly utilized CNFs. Pulp is one of the most abundant forms of bulk cellulose available as natural wood, consisting of randomly oriented nanocelluloses. To prepare a composite material, 30 mg of SWCNT with an average diameter of 1.6 nm and 0.3 g of the pulp was mixed in 30 mL of de‐ionized water (DIW). To structurally change the bulk form to the nanoscale without requiring additional chemicals, sufficient energy is necessary. First, the mixture was subjected to an ultrasonic homogenizer (SONICS, VCX Series) but could not be homogenized, although the temperature of the aqueous mixture increased rapidly. In addition, the pulp and the SWCNTs were also separated after dispersing the mixture by using the high‐shear homogenizer (HG‐15D, Daihan) with a mixing speed of 10 000 rpm. Therefore, we applied zirconia beads with an average diameter of 1 mm, which is widely used in the ball‐milling process, as the media to transfer mechanical energy directly into the bulk mixture. When loaded in the high‐power shaking machine, the kinetic energy generated in the zirconia beads was transferred to the pulp and SWCNTs through countless collisions in the aqueous mixture. Based on SEM EDS analysis in Figure S1 (Supporting Information), the frictional process using zirconia beads did not introduce the residual by‐product in the resultant aqueous ink. Subsequently, a homogeneous black solution containing the nano‐scale composite with cellulose and SWCNTs was obtained within several minutes after the shaking process. First, we determined the influence of the mechanical shaking process in the presence of zirconia beads on the pulp only, SWCNTs only, and the mixture of the pulp and SWCNTs. **Figure** **2**a and Figure S2 (Supporting Information) show that 0.1 wt.% of the SWCNTs do not dissolve in water because of their hydrophobic characteristic.^[^ ^3^, ^10^ ^]^ In the solution with 1 wt.% pulp, the size of the pulp piece decreased as the shaking time (t_shake_) increased. However, the pulp precipitated even in the sample obtained after shaking for 240 min. Although previous studies reported that high‐energy ball‐milling using zirconia grinding balls pulverized a wood‐pulp suspension in water into a homogeneous CNF solution, the presented experimental conditions using the 30 W‐shaker machine to fragment the bleached Eucalyptus hardwood kraft pulp could not introduce the uniform aqueous dispersion based on CNFs.^[^ ^17^ ^]^ By contrast, the mixture of pulp and SWCNTs in water was homogenized after shaking for 20 min. As t_shake_ increased, the uniformity of the resultant solution increased. Moreover, the prepared aqueous ink consisting of cellulose and SWCNTs (C‐CNT) demonstrated a stable dispersion property because no precipitant was obtained after placing it at room temperature for a week. Additionally, to confirm the dispersion stability of C‐CNT inks, zeta (ζ)‐potentials of an aqueous solution based on SWCNTs only and C‐CNT ink were measured with the electrophoretic light scattering method. As shown in Table S1 (Supporting Information), C‐CNT ink exhibited a negative potential enough to keep the dispersion colloidally stable, although the solutions with SWCNTs had only −3.59 mV of ζ‐potential.^[^ ^3d^ ^]^ Nanoparticles dispersed in a solution with a high ζ‐potential are electrically stabilized, while the suspension with a low ζ‐potential of the solution tends to coagulate or flocculate. Especially, the negative ζ‐potential indicates that the dispersed particles are negatively charged. This result showed great consistency with the previous results, where higher negatively charged CNF can disperse more SWCNTs.^[^ ^3d^ ^]^ Moreover, the apparent hydrodynamic diameter calculated from the measured dynamic light scattering data were 1163, 1609, and 2925 nm for C‐CNT inks shaken for 40, 80, and 160 min, respectively. (Figure S3 and Table S2, Supporting Information) These results indicate the generation of nano‐ or micro‐sized particles dispersed in C‐CNT inks after the shaking process. Subsequently, we tested the electrical characteristics of the prepared C‐CNT ink obtained under various t_shake_ values. Because the concentrations of the cellulose and SWCNTs were identical regardless of the preparation step used for obtaining the C‐CNT inks, we dropped 0.3 mL of the C‐CNT ink onto a 20 × 20 mm^2^ glass substrate for ease of comparison. As shown in Figure S4 (Supporting Information), the phases of the drop‐casted sample obtained using the 20‐min‐shaken solution were separated into water and the C‐CNT mixture, resulting in a non‐uniform film after completely drying in a convection oven at 120 °C.

In contrast, the C‐CNT inks shaken over 40 min demonstrated no phase separation after drop casting and uniform C‐CNT films were obtained after the drying process. The rectangular symbol in Figure 2b corresponds to the average of the measured sheet resistance values corresponding to various durations of shaking. As t_shake_ increased from 40 to 240 min, the electrical sheet resistance increased exponentially from 15.5 to 1390 Ω sq^−1^. Using the thickness measurement from the cross‐sectional scanning electron microscope (SEM) images in Figure S5 (Supporting Information), we calculated the electrical conductivities of the C‐CNT films. The total electrical characteristics are summarized in Table S3 (Supporting Information). As shown in Figure 2b, the average conductivity of the 40‐min‐shaken ink is 102.4 S cm^−1^, and that of the best sample is 111.0 S cm^−1^. To the best of our knowledge, this is the highest reported conductivity of a polymer‐matrix nanocomposite based on 9 wt.% carbon nanomaterials.^[^ ^1^, ^3^, ^4^, ^8^, ^9^, ^11^, ^12^, ^18^ ^]^ Furthermore, the electrical conductivity changed gradually with the changes in t_shake_ owing to the internal structure of the prepared C‐CNT composites having a strong correlation to the preparation step that does not require any chemical additives.

To analyze the crystalline structure of the C‐CNT films, we studied X‐ray diffraction (XRD) patterns, as XRD is a well‐established and powerful macroscopic structural characterization tool. We characterized the drop‐casted film with only the SWCNT shaken for 80 min, only cellulose shaken for 80 min, and C‐CNTs obtained under various t_shake_ values. As shown in Figure 2c, cellulose shows two major peaks at 2θ = 16.5° and 22.4° corresponding to the (110) and (200) lattice planes, respectively, which suggests that a typical cellulose I crystalline form is present.^[^ ^8^, ^14^, ^16^ ^]^ Because parallel‐packed cellulose I is the most abundant cellulose form found primarily in the plant cell wall, the XRD peaks confirmed that no significant structural changes occurred while mechanically shaking the natural pulp in water.^[^ ^8^, ^14^ ^]^ Additionally, the calculated index of crystallinity (CrI) of the cellulose film shaken for 80 min was ≈11.1%, in which ≈88% of the fibrillar cellulose was less ordered or amorphous. The XRD peaks from cellulose I in the C‐CNT films were also located at the same positions, which indicates that the added SWCNTs did not affect the crystallinity of cellulose in the aqueous mixture. In contrast, the XRD patterns of the SWCNTs showed an outstanding variation between the SWCNTs only and the C‐CNT films. In the XRD pattern of the bare SWCNTs, the peaks at 2θ = 43.5° and 44.5° were assigned to the (100) and (101) planes, respectively, which reflects the structure of an individual CNT.^[^ ^19^, ^20^ ^]^ Furthermore, the (002) peak at a small angle of ≈2θ = 26.0° reflects a structure resulting from the contributions of both an individual CNT and the ensemble.^[^ ^20^ ^]^ Surprisingly, the outstanding decrease of the intensity at (100) and (101) peaks was observed in the XRD patterns of the C‐CNT composites, even for those obtained under small t_shake_ values. XRD patterns in Figure S6 (Supporting Information) indicated that the shaking process for only 2 min introduced a dramatic decrease of the peak intensities and that C‐CNT films with t_shake_ over 5 min showed negligible change at (100) and (101) peaks. Additionally, the increase in the intensity of the (002) peak was pronounced as increasing t_shake_, and the peak positions were shifted to large angles (refer to the inset graph in Figure 2c and Figure S6 (Supporting Information). The XRD patterns of the CNTs have some distinct similarities to those of graphite, probably because of the intrinsic graphene‐like properties of the CNTs. When the SWCNTs are in a bulk form, a bundled network can easily be prepared from the assembled SWCNTs. Especially, the (110) and (101) peaks observed at large angles (2θ > 40.0°) reflect the atomic structure of the graphene sheet in the bundled SWCNT structure.^[^ ^20^ ^]^ It has been reported that SWCNTs can be dispersed in the presence of the charged cellulose media and apparently with more efficient separation of the nanotubes from their bundles.^[^ ^3d^ ^]^ Based on the (110) and (101) peaks that unexpectedly decreased, we suggest that a distinct change occurred in the SWCNT network, and a bundle of SWCNTs was separated into individual SWCNTs during the formation of the C‐CNT ink. Then, the separated SWCNTs did not re‐aggregate into their bundles but individually participated in the additional bonds to cellulose chains.^[^ ^3a,d^ ^]^ Moreover, the changes in the (002) peak represented the inter‐tube contribution changes depending on the aggregate form. In previous studies, the XRD patterns of randomly oriented SWCNTs contained a (002) peak that differed from the one present in the XRD patterns of the aligned samples, with a peak shift to a small 2θ angle because the d‐spacing distributions should be relatively narrow for aligned samples but broad for random samples.^[^ ^20^, ^21^ ^]^ The same trend was observed in the (002) XRD patterns obtained from the C‐CNT ink regardless of the t_shake_ value. Subsequently, we emphasize that the separated SWCNTs from the bundled network participated in the generated well‐aligned network in the C‐CNT during the shaking process.

To investigate the generation of additional chemical bonds during the mechanical shaking process, the Fourier transform infrared (FTIR) spectra of the SWCNTs, bare cellulose film, and C‐CNT films obtained under various t_shake_ values were analyzed. As shown in Figure S7a (Supporting Information), no significant change in the FTIR spectra of bare cellulose films from Eucalyptus pulp was observed under increasing t_shake_, where the peaks at 1026 and 1165 cm^−1^ represent the C−OH stretch and C−O−C antisymmetric stretch of primary alcohol or ester in a cellulose molecule.^[^ ^1a^ ^]^ The peaks in the range of 1270–1600 cm^−1^ were the absorption bands of the residual kraft lignin in pulp.^[^ ^23^ ^]^ Furthermore, the FTIR spectra obtained from C‐CNT films in Figure S7b (Supporting Information) show almost identical peaks to that of the cellulose film regardless of a t_shake_.^[^ ^1a^ ^]^ However, two absorption peaks at 2910 and 3320 cm^−1^ increased as increasing t_shake_, corresponding to the vibration of the hydroxyl group and the stretching vibration of the methylene group, respectively. These results indicate that there was no outstanding change in the chemical structure of pulp and that the additional hydroxyl groups on the surface of SWCNTs were introduced during the shaking process in the presence of water.^[^ ^1^, ^3^, ^19^ ^]^

To confirm the interaction between cellulose and the SWCNTs in the C‐CNT composites, the Raman spectra of the SWCNTs and the C‐CNT films obtained under various t_shake_ values were carefully studied. The Raman spectra in the range of 800–2000 cm^−1^ are dominated by two characteristic peaks from the SWCNTs: the G‐band at 1600 cm^−1^ attributed to the in‐plane vibration of the C−C bonds and the D‐band at 1350 cm^−1^ induced by the disorder owing to the defects and curvature in the nanotube lattice.^[^ ^19^, ^22^ ^]^ As shown in Figure 2d, the Raman spectra of all the C‐CNT films showed radial‐breathing‐mode (RBM), G‐ and D‐ bands, which were present in the spectrum of the tested SWCNT samples. The peak position of the G‐band in the Raman spectra of the SWCNT sample only was 1592.2 cm^−1^, and the Raman spectra of the C‐CNT films obtained under t_shake_ values of 20, 40, 80, and 240 min showed G‐band peaks at 1595.9, 1597.7, 1595.9, and 1597.7 cm^−1^, respectively (refer to the right inset of Figure 2d and Figure S8, Supporting Information). Changes in the peak position of the G‐band owing to the C−C bonds in the SWCNTs are strongly related to the vibrating carbon atoms surrounding the SWCNTs.^[^ ^22^ ^]^ The up‐shift in the G‐band position suggests that strong non‐covalent interactions such as hydrogen bonds or van der Waals bonds between nanocelluloses from cellulose and individual SWCNTs were created.^[^ ^3^, ^22^ ^]^ Additionally, the RBM peaks of SWCNTs in Figure S8c (Supporting Information) changed before and after they were incorporated into cellulose. Because the RBM is sensitive to changes in the surrounding chemical environment, the RBM of the C‐CNT films indicates that the SWCNTs were noncovalently incorporated into cellulose chains. Moreover, the integral area ratios of the D‐ and G‐bands (I_D_:I_G_) can be used to evaluate the extent of any carbon‐containing defects. The inset graphs in Figure 2d indicate the gradual increase in the peak intensity of the D‐band (1350 cm^−1^) corresponding to the C‐CNT samples with increasing t_shake_. In contrast, the intensity of the G‐band (1600 cm^−1^) remained constant.

Additionally, the calculated I_D_:I_G_ of the C‐CNT films changed from 1:44 (t_shake _= 20 min) to 1:13 (t_shake _= 240 min), whereas the I_D_:I_G_ of the bare SWCNTs was 1:56 (t_shake_ = 0 min). This noticeable change in the I_D_:I_G_ of the C‐CNT films indicates that the number of carbon‐containing defects gradually increased with increasing t_shake_s. This result is consistent with the FTIR spectra in Figure S7b (Supporting Information), indicating the gradually increasing contents of hydroxyl groups and methylene groups in C‐CNT films with increasing t_shake_s. Because disordered carbon atoms can be generated at the broken sites in the nanotube lattice, we estimated that some of SWCNTs were destroyed during the manufacturing procedure owing to the collisions of the heavy zirconia beads.^[^ ^22^, ^24^ ^]^

In addition, the morphologies of the dried C‐CNT films clarified the structures of the nanocomposites in the tested C‐CNT inks. Figure 2e and Figure S9 (Supporting Information) present the 3D surface morphological images of the C‐CNT films corresponding to various t_shake_ values obtained using the optical surface profiler. The average value of the measured surface roughness over 461 × 612 µm was 0.43 (±0.07), 0.44 (±0.04), 0.67 (±0.14) and 0.77 (±0.07) µm of the C‐CNT films obtained under t_shake_ values of 40, 80, 160 and 240 min, respectively. The longer the shaking time during the preparation step of the C‐CNT ink, the rougher the surface of the C‐CNT film. Moreover, the SEM images clearly observed the variation of the structural morphology of the C‐CNT films with increasing t_shake_. As represented in Figure S10 (Supporting Information), nanowires with diameters in the range of 20–30 nm are developed until t_shake_ reaches 40 min, and the surface of the C‐CNT film is covered with the entangled structure consisting of lots of nanowires. Notably, the generated morphology with developed nanowires was considerably different from those of bare cellulose and the SWCNTs only (FigureS11, Supporting Information).

Additionally, the formation of the nanowires and their entangled network could not be adequately explained by the simple mixture or composite of cellulose and the SWCNTs. Whereas, at t_shake_ values of over 80 min, the characteristic nanowire entanglement started to be destroyed, the diameters of the nanowires began to increase, and other aggregated compounds began to fill the inter‐fiber region. We emphasize that this distinguishable morphological change at the surface of the C‐CNT films correlates with the change of surface roughness in the 3D surface morphological images displayed in Figure 2e. Using the following high‐resolution SEM images of the C‐CNTs in Figure 2f, we can confirm the stepwise change in both the developed nanowires and the resultant structural morphology of the C‐CNT films with increasing t_shake_ values. At a t_shake_ of 20 min, the nanowires began developing from the nonfibric compound, and the generated nanowires remained attached to the original compound. At a t_shake_ of 40 min, the nanowires with a diameter of 20 nm entangled with each other, forming a network structure. At a t_shake_ of 80 min, a filling in the inter‐fiber region was observed with the increase in the diameters of the nanowires. Finally, the aggregated structure with nanowires thicker than 100 nm was developed at a t_shake_ of 160 min. The diameters of the developed nanowires under various t_shake_ values were estimated using the transmission electron microscope (TEM) images in Figure 2f.

Additionally, the TEM images in Figure S12 (Supporting Information) reveal that the developed nanowires consisted of alternative chains represented by repeating white and black lines. In previous studies using the same SWCNTs, the white and black lines in TEM images corresponded to the empty space inside a SWCNT and the carbon walls of a SWCNT, respectively.^[^ ^18a^ ^]^ At the same time, when a SWCNTs were coated or surrounded by a polymer material, the polymer material was also shown as alternating black lines.^[^ ^18b^ ^]^ The high‐magnification TEM images in Figure S13 (Supporting Information) reveal that the nanowires in the 40‐min‐shaken C‐CNT ink have diameters in the range of 20.2–24.3 nm. Because the average gap between the black and white lines was 0.88 (±0.10) and 1.30 (±0.11) nm, respectively, we concluded that the white lines correspond to the individual SWCNTs and the black area between the SWCNTs filled with nanocellulose consists of 2 or 3 molecular cellulose chains.^[^ ^18^, ^25^ ^]^ In the C‐CNT ink obtained using a t_shake_ of 40 min, 10–12 highly aligned SWCNTs were covered with nanocellulose chains with a uniform thickness. As t_shake_ increased, the developed nanowire maintained the alternating structure with increasing numbers of highly aligned SWCNTs. We emphasize that the formation of nanowires consisting of highly aligned SWCNTs in a cellulose matrix is reported for the first time for a specific nanocomposite structure based on a reinforcing carbon nanomaterial and a polymer matrix, which can explain the highest electrical conductivity of the conductive C‐CNT ink.

The developed internal structure of the prepared conductive ink based on the C‐CNT nanocomposites can also affect the mechanical properties of the dried C‐CNT films. The typical tensile stress‐strain curves of the conductive C‐CNT films obtained under various t_shake_, bare cellulose films, and the utilized pulp were evaluated in terms of stress and elongation at break. As depicted in Figure 2g, the prepared C‐CNT and cellulose samples show linear behavior at the first stage until the breaking point is reached, when the stress decreases sharply owing to the broken film along the stretching direction. The data in **Table** **1** reveals that the bare cellulose film and natural pulp possess similar tensile strengths and Young's moduli but dissimilar strains to break owing to the nonuniformity imparted by the 0.3‐mm‐thick pulp. As discussed, the mechanical shaking process using zirconia beads did not influence the internal structures of the nanocelluloses in natural pulp alone, which resulted in the bare cellulose film with a cellulose I crystalline form. The Young's modulus and stress of the C‐CNT film obtained under a t_shake_ of 40 min were noticeably increased. The unique mechanical properties of the SWCNTs, i.e., high tensile strength and modulus, can be used for reinforcing the cellulose matrix; this enhancement can be explained by the property averaging effect in the composite based on cellulose and the SWCNTs, because the physical properties of a composite must, in some way, follow an average of the properties of their individual components.^[^ ^9b^ ^]^ However, the decrease in mechanical properties with increasing t_shake_ from 40 to 240 min cannot solely be explained by the property averaging because the total volume of the SWCNTs used as reinforcement in a C‐CNT composite was almost constant irrespective of t_shake_. In Krenchel's approach for determining the mechanical properties of a composite, the shape, orientation, and length of fillers contribute to the strength along the axis of the applied force.^[^ ^9^, ^24^ ^]^ As discussed, the SWCNTs were considerably reduced in length during the manufacturing of the C‐CNT inks in water. When the lengths of the SWCNT in the C‐CNT composite were sufficient, the destruction of the C‐CNT composite proceeded with the rupture of the nanotubes. However, the reinforcement for the same volume fraction was reduced for short SWCNTs because the degree of non‐covalent adhesion between the cellulose matrix and the individual SWCNT weakened. Nevertheless, the sudden reductions in Young's moduli and tensile strengths of the C‐CNTs obtained under t_shake_ values of 120 and 240 min were not clearly understood using Krenchel's approach.^[^ ^18b^ ^]^ Moreover, the dried C‐CNT films became increasingly brittle as t_shake_ increased, which indicated that the ductility of the C‐CNT samples decreased along with the stepwise decrease of strain to fracture. These results indicate that additional structural changes in the C‐CNT nanocomposites are possible during the mechanical shaking process, which is highly related to the size of the nanowire and the internal network structure of the developed nanowire entanglement. In the pre‐existing crack model, tiny cracks with several hundred inter‐atomic distances can be frequently formed during the manufacturing of a material or as a result of mechanical processes.^[^ ^26^ ^]^ As pre‐existing cracks, these structural changes can be attributed to the resultant brittle nature of C‐CNT inks with high t_shake_.

**Table 1:** Mechanical and electrical properties of natural pulp, the cellulose film obtained after shaking natural pulp in water, and the C‐CNT nanocomposites obtained under various shaking times. Some results from previous studies with various CNT contents are listed for comparison.

	Contents of SWCNTs	Young's modulus	Tensile strength	Strain to fracture	Conductivity
Natural pulp	−	10.1 MPa	15.7 MPa	4.11%	−
Cellulose (80 min)	−	11.5 MPa	19.2 MPa	2.84%	−
C‐CNT (12 min)	1 wt.%	−	−	−	2.9 S cm^−1^
C‐CNT (20 min)	4.8 wt.%	−	−	−	26.2 S cm^−1^
C‐CNT (40 min)	9 wt.%	44.5 MPa	51.2 MPa	2.66%	102.4 S cm^−1^
C‐CNT (80 min)	9 wt.%	39.2 MPa	37.9 MPa	1.80%	34.2 S cm^−1^
C‐CNT (120 min)	9 wt.%	18.1 MPa	12.0 MPa	0.78%	17.1 S cm^−1^
C‐CNT (240 min)	9 wt.%	7.2 MPa	4.3 MPa	0.20%	0.9 S cm^−1^
C‐CNT (120 min)	23 wt.%	−	−	−	188.0 S cm^−1^
C‐CNT (240 min)	33 wt.%	−	−	−	362.3 S cm^−1^
CCNB^[a]^	3.5 wt.%	27.0 GPa	330 MPa	2.5%	54.2 S cm^−1^
NFC+SWCNT^[b]^	10 wt.%	11.2 GPa	213 MPa	7.0%	3.23 S cm^−1^
Cellulose+SWCNT^[c]^	10 wt.%	‐	62.0 MPa	4.8%	10.0 S cm^−1^
NFC+SWCNT^[d]^	43 wt.%	14.0 GPa	200 MPa	2.1%	207 S cm^−1^
CNC+SWCNT^[e]^	75 wt.%	14.4 GPa	142 MPa	1.39%	515 S cm^−1^

^a]^ Carbon nanotube‐cellulose nanofiber bulk (CCNB) films by the layer‐by‐layer assembly and hot pressing for densification^[^ ^33a^ ^]^;

^b]^ High‐density nanopapers from the aqueous dispersion consisting of SWCNTs and the carboxy‐methylated nanofibrillated celluloses (NFCs)^[^ ^3a^ ^]^;

^c]^ Casted films in an acidic coagulation bath using the NaOH/urea aqueous mixture consisting of SWCNTs and cellulose^[^ ^9^ ^d]^;

^d]^ Ordered microfibers by injecting the aqueous dispersion consisting of SWCNTs and the carboxy‐methylated nanofibrillated celluloses (NFCs)^[^ ^3a^ ^]^;

^e]^ Hot‐pressed high‐density nanopapers from the aqueous dispersion consisting of SWCNTs and the highly charged cellulose nanocrystals (CNCs).^[^ ^3d^ ^]^

Next, we performed the reusability test using the same C‐CNT sample as that used for measuring mechanical properties. To track the change in the electrical properties depending on the re‐dispersing condition, a completely dried C‐CNT film with an electrical conductivity of 102 S cm^−1^ (t_shake_ of 40 min) was selected (Figure S14a, Supporting Information). Pieces of the C‐CNT samples were loaded in DIW to replicate the concentration of the initial C‐CNT ink. Although the mixture was stirred continuously for a week, no significant change was observed, except for the wetting of the C‐CNT samples (Figure S14b, Supporting Information). Despite subjecting the mixture with zirconia beads to an ultrasonic homogenizer, the solution could not be homogenized (Figure S14c, Supporting Information). In contrast, we obtained a re‐dispersed aqueous C‐CNT ink using a high‐power shaking machine with an additional 10–40 min shaking time. All reused inks were uniform colloidal solutions similar to the initially prepared C‐CNT ink (Figure S14d,e, Supporting Information). We also checked the electrical conductivities of the drop‐casted films after they were fully dehydrated. The electrical conductivity of the reused inks reduced as the additional t_shake_ of the re‐dispersed solution increased (Table S4, Supporting Information). Notably, if the accumulated shaking time (40 min + additional t_shake_) for the reused C‐CNT ink was equal to the t_shake_ used for the pristine C‐CNT ink, both C‐CNT films exhibited similar electrical conductivities. This result confirms that the proposed conductive C‐CNT ink can be reused after mechanical shaking, although its electrical conductivity degrades owing to additional shaking time.

**Figure 3:**
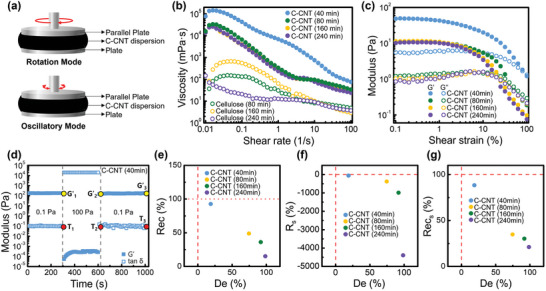
a) Schematic illustration of the rheological measurement mode using the C‐CNT suspension ink, b) steady shear viscosity as a function of shaking time of cellulose (open symbols), and the C‐CNT with 0.1 wt.% of SWCNT (solid symbols) suspensions. c) Amplitude sweep test as a function of shaking time (solid symbols are G’ and open symbols are G”), d) 3 interval thixotropic tests for evaluating the coating property of the C‐CNT (40 min) suspension, and phase diagrams showing e) *De* versus *Rec*, f) *De* versus *Rs*, and g) *De* versus *Recs* of the C‐CNT suspensions according to shaking time.

To analyze the stepwise development of the nanowires in the conductive C‐CNT inks formed by the mechanical collision process, the rheological properties of both the cellulose suspensions and the C‐CNT inks were investigated in the rotational and oscillatory shear modes (**Figure** **3**a). Mechanical forces generally disintegrate the network structures of celluloses, ultimately reducing them to the nanoscale.^[^ ^27^ ^]^ The rheological behavior of celluloses is subject to several influences, including the specific type of cellulose used, the microstructural characteristics of the dispersion, and the processing conditions. Collectively, these factors determine the viscoelastic properties and overall performances of the cellulose‐based suspension under investigation.^[^ ^28^ ^]^ Regardless of the duration of shaking, the samples display a clear shear‐thinning behavior in response to increasing shear rates, as shown in Figure 3b. The observed reversal in the viscosity of cellulose, which is correlated with shaking time, can be attributed to the flow‐induced rearrangement of the cellulose fibers. Additionally, the disappearance of the overshoot at low shear rates with the increase in shaking time may be attributed to an imbalance between the rates of destruction and generation driven by the cellulose fibrils by the mechanical force‐induced nanonization.^[^ ^29^ ^]^ Moreover, incorporating the SWCNTs into cellulose solution significantly increased viscosity by an approximate factor of 100 and induced the emergence of a plateau region for C‐CNT dispersion with a t_shake_ over 40 min. The appearance of this plateau region is related to the formation of nanowires from the mixture of pulp and SWCNTs. During the preparation process shown in Figure 1, we estimate that the SWCNTs associated with the cellulose fibrils, splitting the bulk cellulose and eventually forming nanoscale composites of cellulose and SWCNTs, which disperses the developed nanowires in the C‐CNT ink. This suggests a transformation in an arrangement of nanowires facilitated by the flow, particularly discernible around a shear rate of 1 s⁻¹. In addition, the storage (G') and loss (G“) moduli of cellulose and the C‐CNT inks were evaluated via small‐amplitude oscillatory shear (SAOS) testing in the oscillatory mode (The lower image in Figure 3a), where SAOS testing can elucidate the linear viscoelastic characteristics of complex fluids.^[^ ^30^ ^]^ Regardless of shaking time, cellulose consistently demonstrates a low modulus, displaying a solid‐like behavior (G' > G”), as shown in Figure S15 (Supporting Information). However, for the developed nanowires in the C‐CNT ink, a pronounced enhancement in the modulus was discernible (Figure 3c). Additionally, the C‐CNT manifested a crossover point between the G' and G“ with increasing shear strain. Interestingly, the crossover point of G' and G” indicates that the shear strain decreases from 100% to 20% with increasing shaking time, implying a degradation of structural stability over time. With increasing shaking time, an overshoot phenomenon became apparent in the G“, highlighting the structural rearrangements within the C‐CNT inks upon deformation. To confirm that the structure of C‐CNT inks has been further developed depending on a t_shake_ through the formation of incorporated SWCNTs into cellulose fibers, the oscillatory frequency sweep measurements were conducted to analyze the C‐CNT ink structure retention after ink deposition. In Figure S16 (Supporting Information), frequency sweep measurements were carried out in the frequency range of 0.1 to 100 rad s^−1^ at a constant strain of 0.1%. The tan δ (G”/G') value of the C‐CNT suspensions (80–240 min) is larger than C‐CNT (40 min) at the low frequency (<3 rad s^−1^). This indicates that C‐CNT suspensions (80–240 min) have good flowability at low frequencies. However, at high frequencies (>3 rad s^−1^), the opposite tendency is indicated, and the suspensions except for C‐CNT (40 min) are predominantly elastic. Consequently, the flowability of all C‐CNT suspensions is generally good at low frequencies, except for C‐CNT (40 min) which exhibits good flowability at high frequencies. Therefore, the t_shake_ can influence the internal structure of C‐CNT inks and determine the printing conditions to be used.

This observed behavior corroborates that the 40‐min‐shaken C‐CNT ink effectively separates the nanocellulose matrix into well‐dispersed nanowires by reinforcing individual SWCNTs. Furthermore, as t_shake_ increased, the sizes of the resultant nanocomposites gradually increased owing to the aggregations of the fragmented nanowires obtained from the collision process (Figure 2f; Table S2, Supporting Information).

To determine the applicability of the aqueous C‐CNT dispersions as a conductive ink in the conventional coating and printing processes, three interval thixotropic tests (3ITT) were conducted using C‐CNT inks based on different t_shake_ values. The 3ITT data shows the deformation and recovery behaviors of each testing sample (Figure 3d; Figure S17, Supporting Information). In these experiments, the shear stress was partitioned into three distinct regions, i.e., 0.1, 100, and 0.1 Pa, at a fixed frequency of 10 Hz. The 3ITT method can be employed to assess the impacts of shear stress and shear rate on the deformation and recovery of a material in relation to applied force. If the ink does not have recovery properties after coating and before the solvent is dried, the material undergoes a structural deformation that may change the coating thickness or other coating properties. In this study, we quantitatively analyzed the structural deformation and time‐dependent structural recovery using the 3ITT measurements, which provide the degree of structural deformation *De*, degree of structural recovery *Rec*, relative solidity *R_s_*, and degree of recovery for relative solidity *Rec_s_*.^[^ ^31^, ^32^ ^]^ **Table** **2** lists these quantitative parameters extracted from the 3ITT data of the storage modulus and tan δ values used for evaluating the coating characteristics of the C‐CNT dispersions. The efficiency of the coating characteristics of the C‐CNT inks can be evaluated by analyzing phase diagrams that offer insights into structural deformation and recovery characteristics.^[^ ^33^ ^]^ Here, *De* signifies the deterioration of the original structure. The extent of structural regeneration in the aqueous C‐CNT dispersions can be assessed using the *Rec* < 100% data in Figure 3e. Among all the samples, the C‐CNT ink subjected to a t_shake_ of 40 min exhibited the least structural collapse and demonstrated optimal structural recovery characteristics. As shown in Figure 3f,g, the solidity of the samples markedly decreases with increased shaking time, evident from the corresponding decrease in the *R_s_* and *Rec_s_* values. These observations suggest that the preparation step involving the collisions of zirconia enhances the performance of an ink property through the formation of well‐dispersed composite nanowires. However, excessive shaking leads to the degradation of the modulus, solidity, structural recovery property, and ink viscosity, limiting the applicability of the C‐CNTs. Consequently, without resorting to chemical treatments, the dispersion and sizes of the developed nanowires in the aqueous C‐CNT ink can be optimized by adjusting the shaking process. In this study, the C‐CNT sample with the highest electrical conductivity of 102 S/cm displayed the most superior coating properties.

**Table 2:** Parameters for structural deformation parameters obtained from three interval thixotropic tests on the C‐CNT suspensions.^[^ ^33^ ^]^

Structural deformation parameters	Equation	Definition
𝑫𝒆 (%)	$\frac{G^{\prime }1\;\mathrm{at}\;1^{st}\;3\mathrm{ITT}\;--\;G^{\prime }2}{G^{\prime }1\;\mathrm{at}\;1^{st}\;3\mathrm{ITT}}\times 100$	**Degree of structural deformation** (+) *De*: Initial structure is broken down (‐) *De*: Further structure is newly developed
𝑹𝒆𝒄 (%)	$\frac{G^{\prime }3}{G^{\prime }1\;\mathrm{at}\;1^{st}\;3\mathrm{ITT}}\times 100$	**Degree of structural recovery** *Rec* > 100%: New structure is generated *Rec* < 100%: Structure is partially recovered
𝑹𝒔 (%)	$\frac{T1\;\mathrm{at}\;1^{st}\;3\mathrm{ITT}\;--\;T2}{T1\;\mathrm{at}\;1^{st}\;3\mathrm{ITT}}\times 100$	**Relative solidity** (+) *Rs*: Enhanced solid‐like character under stress (‐) *Rs*: Enhanced liquid‐like character under stress
𝑹𝒆𝒄𝒔 (%)	$\frac{T1\;\mathrm{at}\;1^{st}\;3\mathrm{ITT}}{T3}\times 100$	**Degree of recovery for relative solidity** *Recs* > 100%: More solid‐like character *Recs* < 100%: Solid character is partially recovered

**Figure 4:**
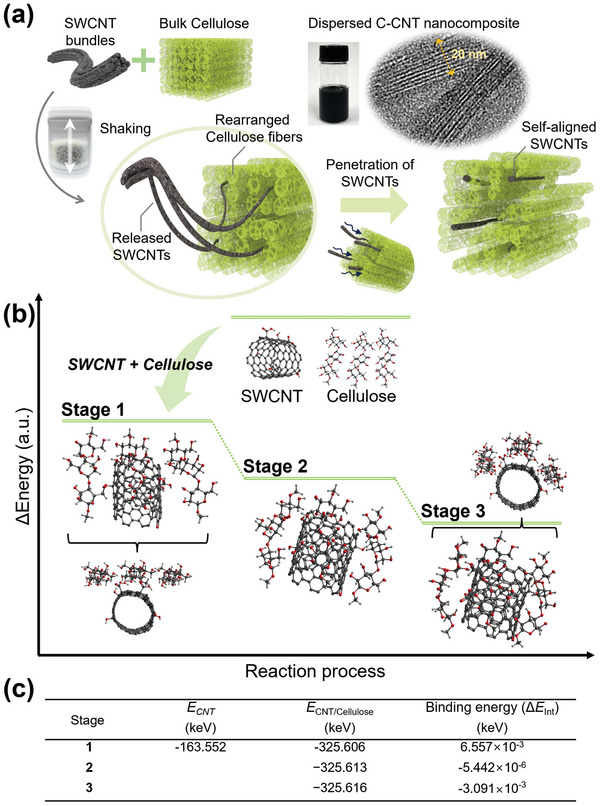
a) Schematic of the mechanism for generating the aqueous dispersion of highly conductive nanowires consisting of self‐aligned SWCNTs surrounded by nanocellulose chains. During mechanical shaking with zirconia beads in water, the cellulose chains in the pulp rearrange owing to the penetration of the released SWCNTs. This results in the alternative chain structure based on C‐CNT. The formation of the C‐CNT nanocomposite splits the bulk cellulose matrix into well‐dispersed C‐CNT nanowires in water. b) Pathways of the formation mechanism of conductive nanowires: Images depict magnified images showing how the celluloses interacted with the nanotube model. c) Calculated interaction energies (*ΔE_int_*) for Stages 1−3 using density‐functional‐theory (DFT) calculations.

Based on the structural analysis of the dried C‐CNT films and aqueous C‐CNT dispersions, we determined the generation mechanism of the conductive nanowires consisting of self‐aligned SWCNTs inside the cellulose matrix (**Figure** **4**a). As mentioned above, the driving force for the specific structural changes in the mixture of natural pulp and SWCNT bundles is the energy imparted by vigorously shaking the zirconia beads together. Specifically, the nanocelluloses in the pulp can be fractured owing to a cascade of hydrogen‐bond breakage and re‐formation events.^[^ ^14^ ^]^ When sufficient external stress is applied, energy breaks the hydrogen bonds between the hydroxyl groups in the molecular cellulose chains. Under the successive external forces, the pulling‐off cellulose segments participate in inter‐fiber sliding but remain bonded to neighboring fibers due to ready re‐forming.^[^ ^14a^ ^]^ It is also known that more energy can be dissipated for the sliding displacement due to the self‐healing nature of hydrogen bonds.^[^ ^14b^ ^]^ Additionally, different from the previous studies, the applied stress onto cellulose fibers during the shaking process is directionless. Resulting of the random collision of zirconia beads, a bare cellulose sample can absorb the imparted energy through repeatable sliding without a noticeable change in the internal structure. (Figures 2c and 3b) In contrast, the energy required to separate an individual SWCNT from a bundle of SWCNTs is much lower than that required to break hydrogen bonds in cellulose chains because SWCNTs are bound to each other through van der Waals forces.^[^ ^3^, ^34^ ^]^ Moreover, the SWCNTs that are separated in water tend to re‐aggregate due to their hydrophobic nature and large specific surface area.^[^ ^3^ ^]^ However, in the case of the aqueous mixture shown in Figure 4a, the transferred energy from the collision of zirconia beads during the shaking process initiates the nanostructural reorganization of the molecular cellulose chains in natural pulp through the breaking and reformation of hydrogen bonds between nanocellulose crystals. From the result of inter‐fiber sliding, the exposed cellulose molecules on the surface can interact with the released SWCNTs, which are electrostatically stabilized through the charges of the cellulose molecules.^[^ ^3^, ^14^ ^]^ As discussed in FTIR measurements, the shaking process in water generates additional hydroxyl groups on the surface of SWCNTs. (Figure S7, Supporting Information) It has also been reported that this association is caused by fluctuations of the counterions on the surface of the exposed cellulose inducing dipoles in the sp^2^ carbon lattice surface of the SWCNTs.^[^ ^3d^ ^]^ Owing to these stabilization effects of the nanocellulose−SWCNT complexes, the subsequent sliding action makes SWCNT associate with the additionally exposed cellulose along with the inter‐fiber sliding direction, which can make the inter‐fiber sliding direction of the cellulose chain opposite to the complex formation. This series of events continues until the entire SWCNT segment participates in the complex generation. Finally, the released SWCNTs penetrate the cellulose matrix following the sliding direction. Similar to the previous reports, the wrapping effect from the surrounded cellulose fibers highly stabilized the nanocellulose‐SWCNT complex and caused the SWCNTs to penetrate the matrix along the inter‐fiber sliding direction of the molecular cellulose chains.^[^ ^3^, ^35^ ^]^ Once the nanocelluloses are rearranged through the inter‐fiber sliding action of the molecular cellulose chains, the released SWCNT segments become stable in the nanocellulose matrix through physical interaction such as van der Waals force, hydrogen bond, and electrostatic stabilization.^[^ ^3^, ^19^, ^34^ ^]^ In addition, the resultant specific alternating structure in the developed composite nanowires can originate from the self‐aligned nature of the settled SWCNTs, because the penetration of a SWCNT segment happens along with the inter‐fiber sliding direction at the interface of the crystalline cellulose I structure. (Figures S12 and S13, Supporting Information) The natural cellulose fibers must structurally rearrange at the parallel‐packed crystalline region like the (110)–(110) interface of the cellulose I β form; this can better explain this phenomenon.^[^ ^14b^ ^]^ This observation is consistent with results obtained from the XRD and Raman analyses in Figure 2 and FiguresS6 and S8 (Supporting Information). In XRD patterns, (002) peak of C‐CNT films shows the increase in intensity and a peak shift to a small 2θ angle, representing the generated well‐aligned network of SWCNTs. The peak shift of the G‐band of SWCNTs in Raman spectra corresponds to the nanocellulose‐SWCNT complex structure where SWCNTs are surrounded by vibrating carbon atoms. In addition, the experimental results reveal that the generated composite nanowires exhibit high‐quality dispersion in aqueous C‐CNT ink, and the entangled nanowires result in a highly conductive film after completely drying. As discussed, the utilized natural cellulose contained an amorphous nanocellulose region of over 80%, which did not participate in the developed nanowires based on self‐aligned SWCNTs. As the nanowire structure developed, neighboring cellulose chains in the amorphous phase split apart because of the relatively weaker binding energy between the cellulose fibers than that between the crystalline ones in the conductive nanowire. The composite nanowires can have several unbounded hydroxyl groups on the surface, which enhances the dispersity of these nanowires in water owing to these cleavages.^[^ ^14^ ^]^

To explain the formation mechanism of conductive nanowires, the reaction pathway was calculated with miniatured models using density functional theory (DFT), as shown in Figure 4b. First, as in Stage 1, the interaction between celluloses and nanotube was unstable due to strong hydrogen bonds between celluloses. However, as in Stage 2, when the celluloses have an overall structure close to the nanotube, the energy value becomes lower and stable. As in Stage 3, the energy value became significantly lowered as cellulose completely surrounded the nanotube. Calculated interaction energies (*ΔE_int_*) for Stages 1−3 in Figure 4c indicate that the structure in which cellulose surrounds the nanotube is thermodynamically stable, and the interaction between cellulose molecules and a SWCNT plays a role in the formation of conductive C‐CNT nanowires.

Based on the proposed generation mechanism of the C‐CNT nanowires, conductive C‐CNT inks with various contents of SWCNTs (3, 15, 90, and 150 mg) in a fixed amount of pulp (0.3 g) were prepared. The optimization of the t_shake_ was conducted to find the maximum conductivity values for each C‐CNT sample with different weight ratios of SWCNTs. As summarized in Table 1, the more contents of SWCNTs in a C‐CNT ink were used, the more t_shake_ was required to make the maximum conductivity. This result indicates that the self‐alignment of SWCNTs can happen through the successive sliding displacement of cellulose chains and that the chance of penetration is not simply proportional to the number of SWCNTs. In addition, it has to be noted that the level of conductivity of C‐CNT films is superior to the previously reported results with the same weight ratio of SWCNTs. (Table 1) The highest measured conductivity was 362.3 S cm^−1^ for C‐CNT ink with 33 wt.% of SWCNTs. Figure S18 (Supporting Information) represents plots of the obtained maximum conductivity of C‐CNT film with respect to the calculated volume fraction of SWCNTs. The solid line in Figure S18 (Supporting Information) corresponds to the critical exponent (p) of the conductivity versus SWCNT volume fraction in the power law percolation theory.^[^ ^3a^ ^]^ In previous works, the value of p is defined as 2 for a 3D random‐network percolation with ideal metal conductors and as 3.146 for a 3D percolation network with a mixture of semiconducting and conducting CNTs.^[^ ^3a,d^ ^]^ However, the conductive nanowires in C‐CNT films showed p of 1.307 and the percolation threshold of the volume fraction almost reached to 0%. Because a C‐CNT film with only 1 wt.% of SWCNTs showed 2.9 S cm^−1^ of conductivity, the additional conducting path is possible such as the entanglement of nanowire composites consisting of the self‐aligned SWCNTs in cellulose. (Figures S5 and S10, Supporting Information)

Furthermore, the morphological analysis and rheological behavior revealed that the gradual size increase of the developed nanowire with increasing t_shake_ originates from the SWCNTs broken during the collision process. Although the SWCNTs demonstrate exceptional mechanical properties, they are brittle and have low impact resistance to transverse stress.^[^ ^24^ ^]^ As discussed using the change of I_D_:I_G_ in Raman spectra with increasing t_shake_s, reinforced SWCNTs can be fractured before and after associating with the cellulose matrix because of the directionless and random collision of zirconia beads. Therefore, two possible routes are possible for fragmenting SWCNTs during the shaking process: 1) simultaneous segmentation, during which the SWCNTs start to break apart at the beginning of the shaking process, or 2) stepwise segmentation, during which the reinforced SWCNTs in a nanowire crack or break. At the early stage of generating the C‐CNT ink, the occurrence of simultaneous segmentation can explain the change in I_D_:I_G_ from the Raman spectra of the C‐CNT ink obtained under a t_shake_ below 40 min, during which the well‐dispersed nanowire structure was not yet developed (Figure 2d). However, based on the results of the reusability tests listed in Table S2 (Supporting Information), we estimate that the stepwise segmentation route is favorable after developing well‐dispersed nanowires in water. This result indicates that the electrical properties of the C‐CNT ink can be further improved by carefully optimizing the shaking conditions.

**Figure 5:**
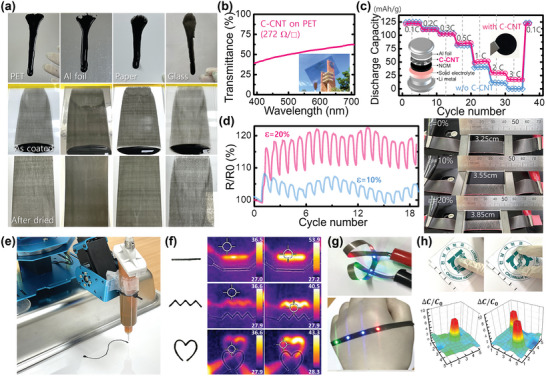
Various applications use the aqueous dispersion of C‐CNT using the conventional coating or printing processes. a) Photographs of the dropped or coated C‐CNT ink on diverse substrates (a PET film, an aluminum foil, an A4 paper, and a glass plate): (top) the flow mark of the dropped C‐CNT ink on an inclined substrate, (middle) the as‐coated C‐CNT ink on a substrate using a bar coater, and (bottom) the dried C‐CNT film on a substrate after bar‐coating. b) Air‐to‐air optical transmittance of the coated C‐CNT film on a PET as a transparent electrode. c) Plot of the discharge capacitance versus current densities of the tested all‐solid‐state batteries (ASSB) at 70 °C with and without the 2 µm‐thick C‐CNT layers coated on aluminum foil as the cathode current collector. The insets show the structure of the tested ASSB (left) and the utilized C‐CNT coating on aluminum foil (right). d) Resistance changes of the C‐CNT layer coated on a stretchable fabric during the stretching test with different strain conditions (ε  = 10% and 20%). The photographs on the right are the tested C‐CNT‐based stretchable electrodes under the corresponding strains. e) Image of drawing line patterns on paper using the lab‐made automotive system to dispense C‐CNT ink. Refer to Movie S2 (Supporting Information) for the entire printing process. f) Photographs and infrared images of the printed C‐CNT electrode on paper. g) Photographs of wearable LED lighting on a glove based on the printed C‐CNT wires on a stretchable tape. h) Photographs of the fully biodegradable force touch sensor arrays based on the printed C‐CNT wires on paper (left) and the corresponding capacitance map of the FTS arrays when touching one or two fingers to the sensor.

A notable feature inherent to the well‐dispersed C‐CNT nanowires in water is the fact that one can utilize the C‐CNT nanocomposite as an electrical element in various electronic applications by using a conventional solution process.^[^ ^1^, ^3^, ^4^ ^]^ From the 3ITT data regarding the deformation and recovery behaviors of the C‐CNT inks and the obtained electrical and mechanical properties of dried C‐CNT films, the proposed C‐CNT ink was found to be a promising and potential alternative to non‐metallic conductive inks. To confirm the compatibility of the C‐CNT ink with the conventional coating and patterning processes, we applied the most conductive C‐CNT ink (t_shake _= 40 min) without using further chemical additives to the conventional bar‐coating and dispenser‐printing processes. First, we checked the wetting of the C‐CNT inks on diverse substrates selected to represent commercially adopted substrate materials such as a Polyethylene terephthalate (PET) film as a plastic, aluminum foil as a metal, an A4 paper, and a glass plate as a ceramic in **Figure** **5**a. When the substrate was inclined after dropping 2 mL of the C‐CNT ink on a surface without any surface treatment, the dropped ink started to flow and left a flow mark. The C‐CNT ink exhibited a good wetting property for all tested substrates because the dropped C‐CNT ink completely filled over the flow mark without any de‐wetted region. The contact angle measurement in Figure S19 (Supporting Information) also confirms that the tested C‐CNT ink shows good wettability on the diverse substrates. This result is consistent with the expected wetting property from the rheological analysis in Figure 3. In Figure 5a, images of the as‐coated C‐CNT ink and the dried C‐CNT films on diverse substrates reveal that the bar‐coating method using C‐CNT inks can be used to prepare a uniform large‐area wet film on diverse substrates. Additionally, no outstanding deformation or strain on the coated ink remained during the conventional drying step.

Next, we fabricated and characterized films for several applications using a coated C‐CNT electrode. The inset image in Figure 5b shows a highly flexible semitransparent electrode (TE) with a C‐CNT film on a PET substrate. The measured sheet resistance was ≈272 Ω sq^−1^, and the average parallel transmittance was approximately 50%. An optical haze meter determined that the C‐CNT showed 61.2% total transmittance and 26.6% optical haze owing to the optical properties of the cellulose film. Particularly, the C‐CNT‐based TE was highly flexible owing to the presence of the entangled nanowires. The flexibility and robustness of the C‐CNT‐based TE were evaluated using a bending test.^[^ ^36^ ^]^ The sheet resistance values were measured after each bending, and the C‐CNT‐based TE film maintained its initial resistance value after bending stress was applied 1000 times under the bending radius of less than 6.5 mm (Figure S20, Supporting Information). Although metallic SWCNTs have significant absorbance in the visible range, the C‐CNT nanocomposite consists of 9 wt.% SWCNT exhibited acceptable optical transparency and electrical properties in relation to highly flexible optoelectronic devices.^[^ ^2a^ ^,b,d,^ ^36^ ^]^ Additionally, we integrated the coated C‐CNT film into a current‐collecting layer in an emerging all‐solid‐state battery (ASSB) device because carbon‐based nanomaterials have been widely used in conventional lithium‐ion batteries.^[^ ^2^, ^37^ ^]^ As shown in Figure 5c, the tested ASSB has a stacked structure with a mixture containing LiNi_0.8_Co_0.1_Mn_0.1_O_2_ (NCM) as a cathode‐active material, a solid‐state electrolyte, and lithium as the anode.^[^ ^38^ ^]^ A 2 mm‐thick C‐CNT layer was prepared on an aluminum foil using the bar‐coating method to form the cathode current collector. To test the influence of the C‐CNT current collecting layer on the electrochemical performances of the ASSBs, we fabricated reference devices using bare aluminum foil as a cathode current collector. The differences in the discharge capacities between the ASSBs with and without the coated C‐CNT layer were not observed at low current rates (C‐rates) in the range of 0.1–0.3 C, as shown in the charge/discharge curves in Figure S21 (Supporting Information), and the discharge capacities in Figure 5c. However, as the C‐rates increased over 0.5 C, the discharge capacities exhibited large differences depending on the cathode material. As listed in Table S5 (Supporting Information), the ASSB with bare aluminum foil exhibits a rapid reduction in its retention ratio from 83.3% to 9.7% when the C‐rate increases from 0.3 to 2 C. In contrast, the C‐CNT current collecting layer increases the retention ratio to 84.3% and 24.8% for the C‐rates of 0.3 and 2 C, respectively. Although the ASSBs without C‐CNT did not show the charge/discharge curve at a high C‐rate of 3 C, ASSBs with C‐CNT still exhibited a discharge capacity of 17.45 mAh g^−1^. The enhanced electrochemical performance originated from the specific nanowire composite structure of the C‐CNT film. Although the electrical conductivity of the C‐CNT layer is much lower than that of aluminum foil, the well‐aligned SWCNTs in C‐CNT and the developed nanowires become 3D current‐collecting sites and efficient conducting paths for electrons.^[^ ^37^ ^]^ Additionally, the electrical loss from the electrical contact at the surface of the coated C‐CNT layer and the aluminum foil was negligible, which is consistent with the efficient film formation on the metallic surface. Further research on C‐CNT as a current collecting layer in ASSB will be reported elsewhere. Moreover, the coated C‐CNT film is promising for preparing electrodes on a stretchable substrate. The curves in Figure 5d present the changes in measured resistance (R/R0) versus the number of cycles of the two strain conditions (ε = 10% and 20%). Although the tested C‐CNT film exhibited the maximum strain value to fracture of 2.84%, the stretchable electrode based on the coated C‐CNT film on a stretchable fabric conducted an electric current at ε = 20%. Additionally, the change in resistance corresponds well to the applied strain conditions during the stretching cycles, despite the sudden increase in R/R0 during the first cycle. Figure 5d reveals the electric current path under the high‐strain condition (ε =  20%). As the applied strain increased above ε = 2.84%, the smooth surface of the C‐CNT electrode cracked. However, unlike the tensile stress‐strain measurement of a single C‐CNT film, the coated C‐CNT electrode did not fracture but maintained connectivity through the cracked surface (refer to Movie S1, Supporting Information). If the stretchable substrate sufficiently withstands the applied stress, the coated C‐CNT electrode can remain electrically conductive at the corresponding strain through deformation of the internal structure. We emphasize that the outstanding stretching property of the C‐CNT electrode can be attributed to both the entanglement of conductive composite nanowires and the strong adhesion on the substrate resulting from the hydrogen bonding of nanocellulose. Owing to the benefits of the coated C‐CNT film as a stretchable electrode, the C‐CNT film can also be applied to force or pressure sensors in wearable devices.

Following the conventional coating process, we checked the compatibility of the prepared C‐CNT inks with the conventional printing process. The dispenser printing process is a widely adopted patterning method in which the amount of ink deposited is controlled by the pressure acting on a syringe and the printing mode used (i.e., droplet or continuous printing). The instrumental setup, including the accuracy of the moving stages, the syringe nozzle size, and the ink's rheological property determines the printing resolution. Using the automotive XYZ positioning robot and a pressurized syringe with a nozzle diameter of 0.6 mm, we set up a dispenser printer that deposits the C‐CNT ink onto a substrate according to the desired printed pattern. As shown in Figure 5e and Movie S2 (Supporting Information), the C‐CNT ink was successfully used as a depositing material in the dispenser printer. Notably, we optimized the process condition to draw line patterns on a paper substrate for eco‐friendly and biodegradable electronic applications. Figure 5f shows the various line patterns prepared by dispensing the conductive C‐CNT ink onto an A4 paper. The prepared line patterns have high adhesion to the paper substrate and, therefore, cannot be removed or damaged owing to simple delamination stress. To confirm electrical conduction, we tested a simple heating element operated via the Joule heating effect. Infrared images revealed uniform conduction along the printed lines. Printing on diverse substrates is also performed on various substrates. For example, we drew several line patterns on a conventional tape for lighting in a wearable application (Figure 5g). Although these lines require a higher voltage than a metallic wire, an array of light‐emitting diodes can be brightly lit without considerable temperature increase using the printed C‐CNT wires; this is beneficial for a wearable device. Another test for a working electronic device involved the use of the printed C‐CNT wires on paper and integration into force touch sensor (FTS) arrays (Figure 5h). The FTS with a 5 × 5 matrix sensor array was prepared by sandwiching an additional 30‐µm‐thick paper between a pair of line‐printed A4 papers using C‐CNT inks. Because the materials used in the FTS are only natural pulp (paper) and SWCNTs, this device is environmentally friendly and biodegradable. Figure 5h represents the curves of the ratio of capacitance change (ΔC/C0) for a single pressure touch sensor.^[^ ^2d^ ^]^ The prepared touch sensor exhibited a high enough sensitivity with a ΔC/C0 of almost 10 when a finger was slightly pressed. Based on the results obtained from various applications involving the conventional coating and printing processes, we emphasize that the aqueous C‐CNT dispersion is a promising non‐metallic conductive ink that is highly compatible with diverse substrates. Additionally, C‐CNT electrodes exhibit outstanding performance and are eco‐friendly, highly flexible, and stretchable, which is beneficial for emerging applications, such as a simple wire, an optoelectronic device, and an energy storage device. As discussed, the device performance can be further improved by increasing the SWCNT content in the conductive nanowire and optimizing the crystalline structure of nanocellulose to improve the SWCNTs' self‐alignment.

## 3 Conclusion

For the first time, we developed a novel and straightforward route to prepare an eco‐friendly, non‐metallic conducting ink based on a highly conductive nanocomposite using natural cellulose and SWCNTs without requiring any chemical treatment or additives. A high‐power shaking machine can generate sufficient impact energy from countless collisions of zirconia beads to induce nanostructural reorganization of the molecular cellulose chains in natural pulp, the release of a bundle of SWCNTs, and the penetration of the released SWCNTs into the cellulose matrix. The resultant cellulose‐SWCNT composites exhibited outstanding development of nanowires, which consisted of alternative chains of well‐aligned individual SWCNTs and nanocellulose with two or three molecular cellulose chains. During the shaking process, both the inter‐fiber sliding action of the molecular cellulose chains with the cellulose I crystalline form and the stabilization of the nanocellulose‐SWCNT complex via physical interactions such as van der Waals force and hydrogen bond caused the self‐alignment of the settled SWCNTs in the nanowires. The structural analysis of the dried C‐CNT films revealed that the entanglement and dispersion of the C‐CNT nanowires determined the mechanical and electrical properties of the dried film. Additionally, the rheological behavior in aqueous C‐CNT inks determined the coating and printing characteristics of the ink. In addition, we identified the generation mechanism of the conductive nanowires from the mixture of natural pulp and SWCNT bundles and the stepwise change of the internal structure of the C‐CNT nanocomposite based on shaking time. In this study, the C‐CNT sample with 9 wt.% of SWCNTs obtained after shaking for 40 min displayed the electrical conductivity of 102 S cm^−1^ and the most superior coating properties. Additionally, the C‐CNT ink with 33 wt.% of SWCNTs showed the highest conductivity of 362 S cm^−1^. Furthermore, the well‐dispersed C‐CNT nanowires in water are highly compatible with the conventional coating and patterning processes. Without using chemical additives or surface treatments, we prepared uniform large‐area electrode films on diverse substrates such as PET, aluminum foil, glass, and paper. In addition, we successfully demonstrated the dispenser printing process that deposits the C‐CNT ink onto a substrate in the desired printed pattern. The high processibility of the C‐CNT ink allowed us to test the coated and printed C‐CNT films for various electronic applications, including a flexible semitransparent electrode film, a cathode current collector in an ASSB, a highly stretchable electrode film, Joule heating wires, electrical wires for a wearable device, and an all‐paper FTS arrays; the inks exhibited superior performance. The industrial demands for eco‐friendly, highly flexible, and stretchable electrodes for emerging applications are increasing; we believe that the aqueous C‐CNT dispersion can be a potential candidate for a highly processible non‐metallic conductive ink compatible with diverse substrates and with high performance. At the same time, it has to be noted that the preparation method of conductive C‐CNT inks by using a high‐power shaking machine is promising compared to the manufacturing technology of conducting composites based on CNFs and CNTs, because the paint shaker used is commercially available and has been widely utilized in the paint industry with the great reproducibility as well as the scalability for the mass production. We also emphasize that the processing conditions using a high‐power shaking machine can be further optimized to increase the electrical conductivity of the C‐CNT inks. This study offers general guidance on preparing a nanocomposite from the structural rearrangement step.

## 4 Experimental Section

### Preparation of the Conductive C‐CNT Ink

Dried Eucalyptus hardwood pulp (NISTRM 8496, Sigma‐Aldrich) as a source of natural cellulose and SWCNTs (TUBALL, the average diameter, and length were 1.3 nm and 5 µm, respectively) were prepared as purchased. The pulp (0.3 g, 0.99 wt.% in a solution) was soaked in 30 mL of DIW and stirred at 25 °C at 200 rpm for 12 h to perform a swelling treatment. Subsequently, the swollen pulp in DIW was sonicated with a tip‐ultrasonic processor (SONICS, VCX Series, 750 W) at 30% power for 5 min. Finally, a powder of SWCNTs (30 mg, 0.099 wt.% in a solution) and 100 g of yttria‐stabilized zirconia beads (CenoTec) with diameters between 0.9–1.1 mm were added to the swollen pulp solution. To prepare the pulp‐only and SWCNT‐only suspensions in water, the same amount of pulp (0.3 g) and SWCNTs (0.03 g) was added into 30 mL of DIW along with 100 g of yttria‐stabilized zirconia beads. Finally, the mixture was physically mixed using a paint shaker machine (SK550 1.1 shaker, 30 W, Maximum operating weight: 40 kg, Fast & Fluid Management) under various shaking times to produce a uniformly dispersed aqueous C‐CNT solution. Details information about optimizing the preparation conditions such as the amount of zirconia beads and the size of zirconia beads can be found in supporting materials. (Figures S22 and S23, Supporting Information) The obtained C‐CNT dispersion was collected in a glass vial by filtering the zirconia beads using a stainless‐steel mesh (d = 0.8 mm).

### Characterization of the C‐CNT Films

To prepare the dried C‐CNT films, the drop‐casting method in which 0.3 mL of the C‐CNT dispersion was dropped onto a 20 × 20 mm^2^ glass substrate using a calibrated micro‐pipet was applied. Subsequently, the drop‐cast films were annealed at 100 °C for 5 min on a hot plate and dried in a convection oven at 120 °C overnight. To determine the electrical properties, the sheet resistance of the C‐CNT film was measured using the van der Pauw method with a digital SMU (Keithley 2400) and subsequently cross‐checked using a noncontact measurement system (20J4, Delcom Instrument, Inc.). The exact film thickness of the C‐CNT film was obtained from the cross‐sectional SEM images (Zeiss Gemini 500 FE‐SEM, Carl Zeiss). Electrical conductivity was calculated using the sheet resistance, and the film thickness was obtained from the SEM measurements. The crystalline structure of the cellulose film, SWCNT film only, and the C‐CNT film were determined from XRD diffractograms (X'Pert PRO MPD, Malvern Panalytical). The Raman spectra of the tested samples were obtained using a laser Raman spectrophotometer (NRS‐5100, Jasco) with a 785‐nm excitation laser. The surface morphological analysis of the drop‐cast C‐CNT film was conducted using 3D profile images obtained via confocal microscopy (NV‐E1000, Nanosystem). The structural morphologies of the C‐CNT films were determined using SEM and TEM images. The SEM (V_acc _= 5 kV) produces high‐resolution images with a magnification of 500000× (a typical resolution power of a few nanometers). For TEM measurements, ≈5 µL of the C‐CNT ink was dropped on a carbon‐coated copper TEM grid (FF200‐Cu, Electron Microscopy Science, USA). The excess solution was removed using filter paper and subsequently dried for analysis. Images were obtained from a 120 kV electron microscope (Tecnai G2, ThermoFisher, USA) and recorded using the Gatan software (Gatan Microscopy Suite 2.0, Gatan, Japan). To analyze the mechanical properties of the C‐CNT film, 20 mL of the prepared C‐CNT inks were loaded in a round‐bottom glass beaker with a diameter of 50 mm, and the water from the C‐CNT ink was slowly evaporated in a convection oven at 120 °C for 8 h. In sequence, the free‐standing C‐CNT film was removed and placed in a vacuum oven at 120 °C overnight for drying completely. The stress‐strain curves were obtained using a tensile test under controlled conditions of 50% relative humidity and 23 °C temperature. Specimens of 50 mm length, 5 mm width, and 10 mm thickness were tested using a universal material testing machine (UTM, Instron 5944) with a 500 N load cell with a crosshead speed of 5 mm min^−1^ corresponding to an initial strain rate of 10% min^−1^. To determine the reusability of the C‐CNT films, 0.154 g of the C‐CNT film subjected to a t_shake_ of 40 min after UTM measurement was soaked in 14 mL of DIW, and 50 g of zirconia beads were added to match the process condition.

### Characterization of the C‐CNT inks

To check the dispersion stability of C‐CNT inks, the zeta (ζ)‐potentials using the electrophoretic light scattering method and the apparent hydrodynamic diameter using the dynamic light scattering (ELSZneo, Otsuka Electronics) was measured. The rheological properties of both the cellulose and C‐CNT coating solutions were quantitatively evaluated by an Anton Paar MCR‐302 rheometer with a 50‐mm‐diameter parallel plate. To determine the behaviors of these solutions under steady shear, the solutions were subjected to strain rates in the range of 0.01–100 s⁻¹. Strain sweep tests were conducted at a fixed frequency of 1 rad s^−1^ with the strain rate varying between 0.1%–100%. The thixotropic behavior was examined via 3ITTs. In these experiments, the shear stress was partitioned into three distinct regions: 0.1, 100, and 0.1 Pa. These evaluations were conducted at a frequency of 10 Hz, and the testing conditions were kept consistent. All measurements were conducted at 25 °C. To avoid solvent evaporation and preserve the composition of the coating solutions, they were sealed with mineral oil during the testing period.

### Bar‐Coating Process Using the Conductive C‐CNT Inks

A conventional wireless bar‐coater (RDS, Bar No. 24) with a wet thickness of 54.86 mm was purchased and cleaned using DIW, acetone, and isopropyl alcohol, successively. To check compatibility, diverse substrates including a 100‐mm‐thick PET film (OHP film, Hyundai office), 20‐ µm‐thick aluminum foil (Wellcos), conventional 20‐mm‐thick A4 paper (Double A), and 0.7‐µm‐thick glass plate (8 × 10 cm, LCD glass) was selected. To maintain the position during the coating process, all the substrates, except the glass plate, were cut into 9 × 10 cm segments and placed on the glass plate using Kapton tape. After blowing the dust and particles away from the surface, a wet film was prepared on the substrate by spreading 3 mL of the C‐CNT ink obtained under a t_shake_ of 40 min using the cleaned bar‐coater. Without requiring any surface treatment such as a plasma or ultraviolet‐ozone treatment, the obtained wet films on the diverse substrates showed excellent coating performance. Subsequently, the wet C‐CNT film on substrates was successively dried in a convection oven at 120 °C and a vacuum oven at 60 °C for 6 h each.

### Calculation of the Interaction Energy During the Formation of C‐CNT

Density‐functional‐theory (DFT) calculations were performed using the ORCA 5.0 program supported by Avogadro software. A simplified carbon nanotube model consisting of 150 carbon atoms was used for the calculations. A cellulose model consisting of 20 carbon atoms and 9 OH functional groups was used for the calculations. The geometry was optimized using the BP86 function with def2‐SVP and def/2J basis sets.

### Characterization of the Flexible TE Film using C‐CNT

Using the wireless bar‐coater (RDS, Bar No. 24), 3 mL of the C‐CNT ink (t_shake_ = 40 min) was coated on a 100‐µm‐thick PET film (OHP film, Hyundai office). After completely drying, the sheet resistance was measured using a noncontact measurement system (20J4, Delcom Instrument, Inc.). Additionally, the parallel‐light transmittance was measured using a fiber‐optic spectrometer (EPP2000, StellarNet. Inc.). The total transmittance and the optical haze value were obtained using an optical haze meter (COH‐400, Denshoku), which yields the average value over the wavelength range of 400–700 nm. Using a lab‐made bending tester, tensile stress was applied to the C‐CNT TE in a bent condition. The change in sheet resistance was recorded against the number of bends.

### Characterization of the Stretchable Electrode using C‐CNT

The stretchable C‐CNT electrode was fabricated by a hot stamping method with thermoplastic polyurethane (TPU) film. A 25 µm thick layer of C‐CNT ink was deposited on a transfer film by bar coating. Then, the transfer film with C‐CNT and TPU was placed on top of a stretchable knitted fabric. The C‐CNT and TPU were thermally transferred to the fabric at 170 °C for 1 minute, resulting in a stretchable C‐CNT electrode.

### Fabrication of the ASSB using C‐CNT as a Current Collector in the Cathode

The electrochemical properties of the C‐CNT current collector were examined using a lithium ASSB. The 80‐min C‐CNT solution was cast onto an aluminum foil and subsequently dried in a vacuum oven at 60 °C for 12 h to prepare a C‐CNT current collector. The electrochemical performance was compared with that of a commercial bare aluminum‐foil current collector as a reference. The ASSB cathode was prepared using an active material (LiNi_0.8_Co_0.1_Mn_0.1_O_2_), a solid electrolyte (Al‐Li_7_La_3_Zr_2_O_12_), Super P (IMERYS Graphite & Carbon, Japan), and a polyethylene oxide (Molecular weight, *M_w_* = 2 × 10 ^5,^ Sigma Aldrich, USA) binder with a stoichiometric ratio of 70:5:5:20. The mixed slurry was cast onto an aluminum foil coated with the C‐CNT composite and subsequently dried at 25 °C for ≈24 h. The starting materials for the solid electrolyte were aluminum‐doped LLZO (Al‐LLZO), polyethylene oxide (PEO), LiClO_4_ (JUNSEI, Japan), and LiFSI (ENCHEM, Korea). First, a lithium complex salt comprising LiClO_4_ and LiFSI in a ratio of 8:2 was prepared by stirring the components in acetonitrile (JUNSEI, Japan) for 30 min. Next, the PEO and the lithium complex salt with a molar ratio of 13:1 were mixed and stirred for 24 h to prepare the PEO‐binder solution. The solid electrolyte (aluminum‐LLZO) and the PEO‐binder solution (PEO, LiClO_4,_ and LiFSI) with a stoichiometric ratio of 70:30 were used. Finally, the mixed slurry was cast onto a PET film and dried at room temperature for ≈24 h to form the solid electrolyte. A 2032 coin‐type cell consisting of the cathode (14ø, loading of the cathode = 5.0 mg cm^−2^), solid electrolyte (19ø), and lithium anode (16ø) was prepared. All the processes were performed in a dry indoor environment. The charge‐discharge measurements of the coin‐type half‐cell were obtained for the potential range of 2.5–4.0 V at different current densities (0.1–3C) using a battery cycler system (WBCS 3000L, Wonatech)

### Dispenser Printing Process Using the Conductive C‐CNT Inks

The dispenser printing system by combining an XY‐plotter robotic arm (Lobot, Bluetooth Control; the arm moving speed was 2 cm ^−1^s) and an air pulse‐dispenser with a nozzle diameter of 0.6 mm (MS‐1D, Musashi engineering Inc.) was set up. The C‐CNT ink (t_shake_ = 40 min) was loaded in the syringe of the dispenser and the amount of ink deposited was controlled by the pressure acting on the syringe. For continuous printing on an A4 paper, 2 bar of N_2_ gas was applied. To print a C‐CNT wire with the desired line pattern, the plotting robot was controlled via an Android app with a Bluetooth connection. After the printing process, the sample was completely dried in a 120 °C convection oven for 6 h.

### Fabrication of the FTS Array using the C‐CNT Wires

To fabricate the FTS with 5 × 5 matrix sensor array, five‐line patterns with a length of 5 cm were printed on a 5 × 5 cm^2^ A4 paper using the dispenser printing setup. The final FTS array was prepared by sandwiching an additional 30‐mm‐thick paper as a dielectric spacer (Shsigma, WP080) between a pair of lines printed on the A4 paper using the C‐CNT inks. The overlapping area between the C‐CNT wires was 2 × 2 mm^2^ for the single sensor. The capacitance values were recorded using an impedance analyzer (Agilent, E4980A) before and after touching a finger.

## Conflict of Interest

The authors declare no conflict of interest.

## Supporting information

Supporting Information

Supporting Information

Supporting Information

## Data Availability

The data that support the findings of this study are available from the corresponding author upon reasonable request.
