# Supplementary material for: Highly Conductive Ink Based on Self‐Aligned Single‐Walled Carbon Nanotubes through Inter‐Fiber Sliding in Cellulose Fibril Networks
Source: Adv Sci (Weinh). 2024 Aug 28;11(40):2402854. doi: 10.1002/advs.202402854 (PMC11516057; doi:10.1002/advs.202402854)
Supplement: Supplementary file 1 — Supporting Information [file ADVS-11-2402854-s001.docx]

Supporting Information

Highly Conductive Ink Based on Self-Aligned Single-Walled Carbon Nanotubes through Inter-Fiber Sliding in Cellulose Fibril Networks

Sejung Park^a^, Yeeun Song^a^, Boeun Ryu^a^, Young-Woong Song^b,c^, Haney Lee^a^, Yejin Kim^a^, Jinsub Lim ^c^, Doojin Lee^a^, Hyeonseok Yoon^a^, Changkee Lee^d^*, and Changhun Yun^a^*

^a^ School of Polymer Science and Engineering, Chonnam National University, Gwangju 61186, Republic of Korea
^b^ Department of Materials Science and Engineering, Chonnam National University, Gwangju 61186, Republic of Korea
^c^ Korea Institute of Industrial Technology (KITECH), Gwangju 61012, Republic of Korea

^d^ Korea Institute of Industrial Technology (KITECH), Ansan-si 15588, Republic of Korea


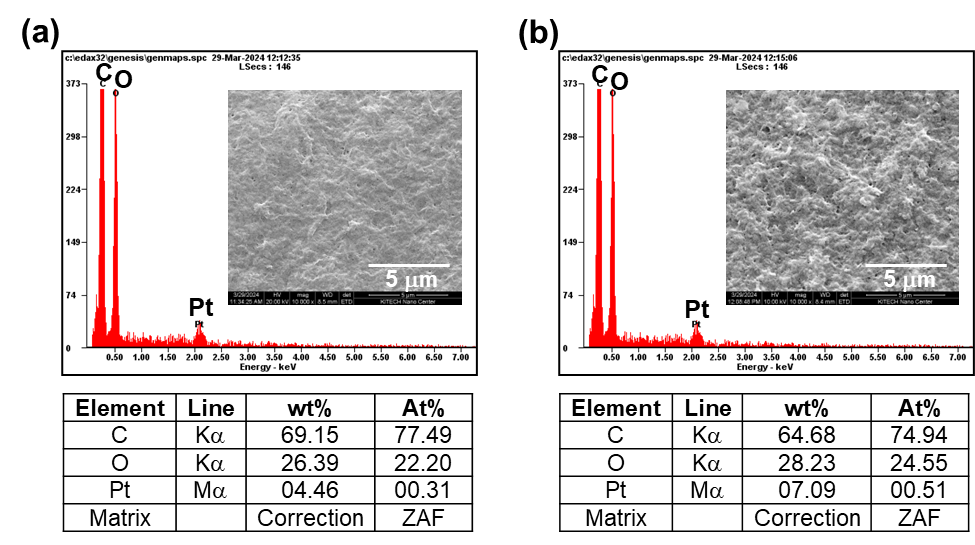


**Figure S1.** SEM EDS analysis of the drop-casted films using prepared highly conductive ink based on cellulose-single walled carbon nanotubes (C-CNTs) with the shaking time of (a) 40 minutes and (b) 240 minutes. For SEM measurement, all samples were coated with thin Platinum (Pt) layer. The lower tables represent the calculated weight percentage for the detected elements.


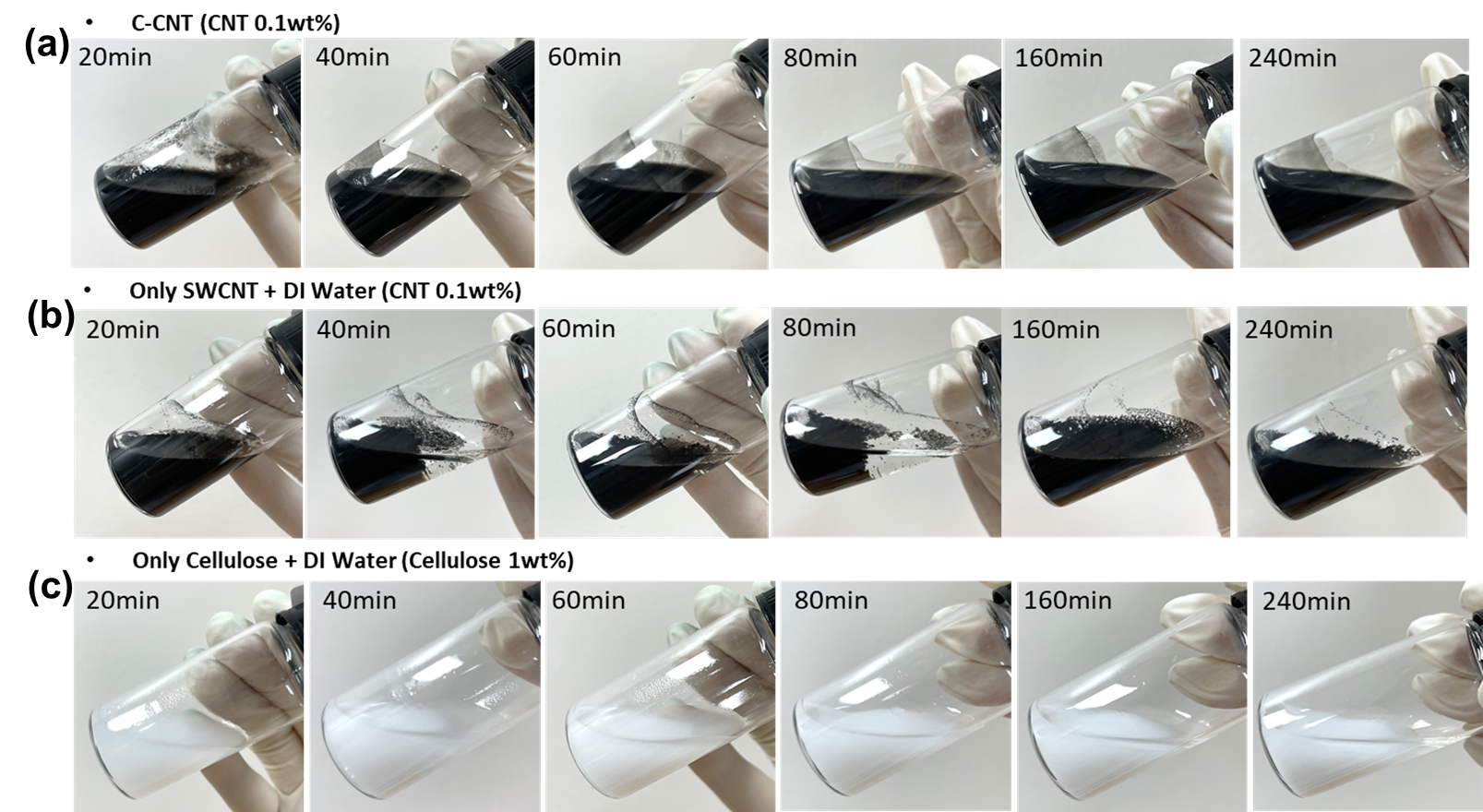


**Figure S2.** Photograph of aqueous suspensions in de-ionized water based on (a) only pulp, (b) only SWCNTs, and (c) the mixture of pulp and single walled carbon nanotubes (SWCNTs) as increasing the shaking time from 20 minutes to 240 minutes.


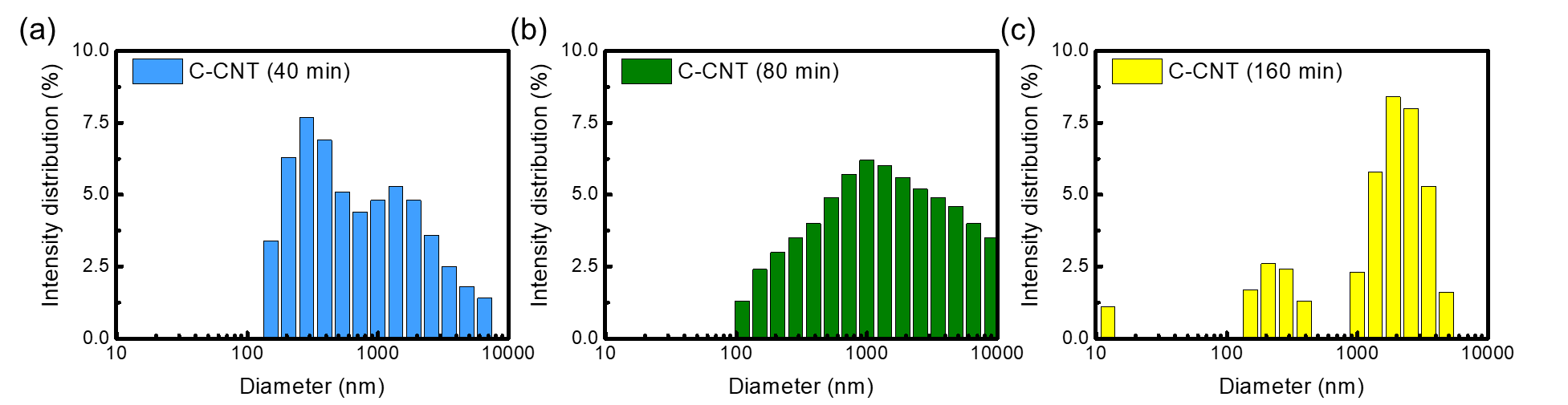


**Figure S3.** The intensity distribution of particle sizes from the measured dynamic light scattering results. The aqueous dispersion of C-CNT was prepared after shaking for (a) 40 min, (b) 80 min, and (c) 160 min.


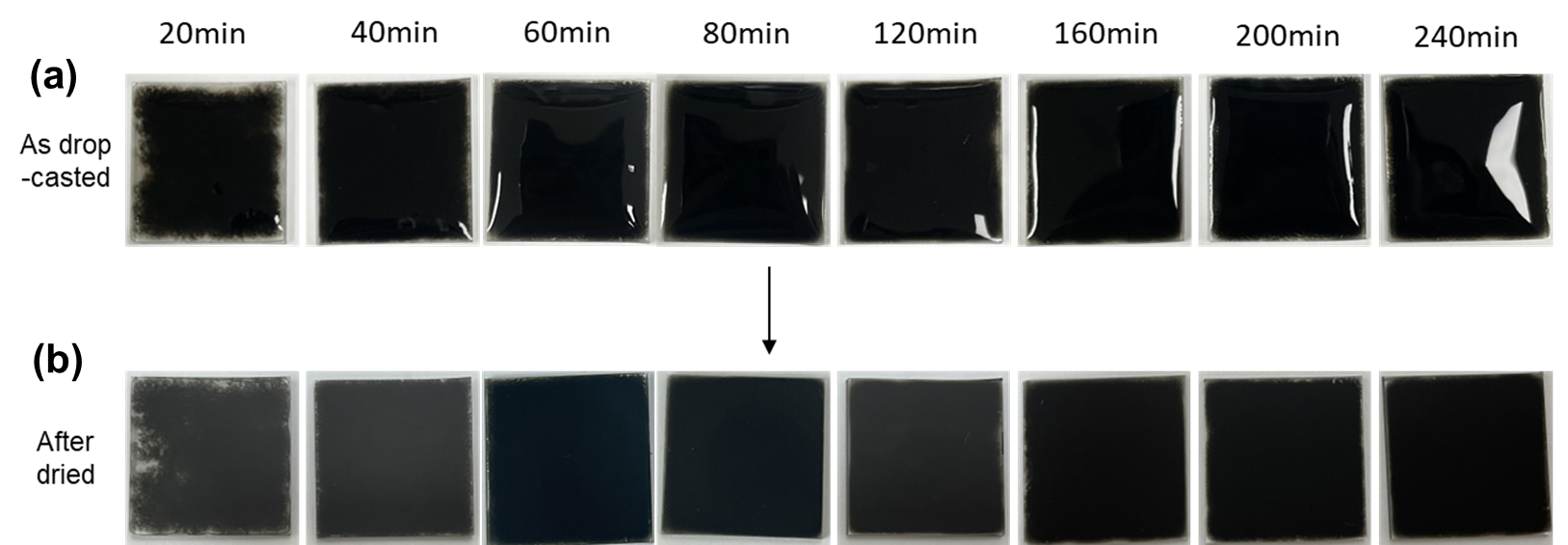
**Figure S4.** Photograph of the drop-casted films using prepared highly conductive ink based on cellulose-single walled carbon nanotubes (C-CNTs) as increasing the shaking time from 20 minutes to 240 minutes. Pictures are taken (a) just after drop-casting 0.3 ml C-CNT ink on a 20 × 20 mm^2^ glass substrate and (b) after completely drying the drop-casted film.

**
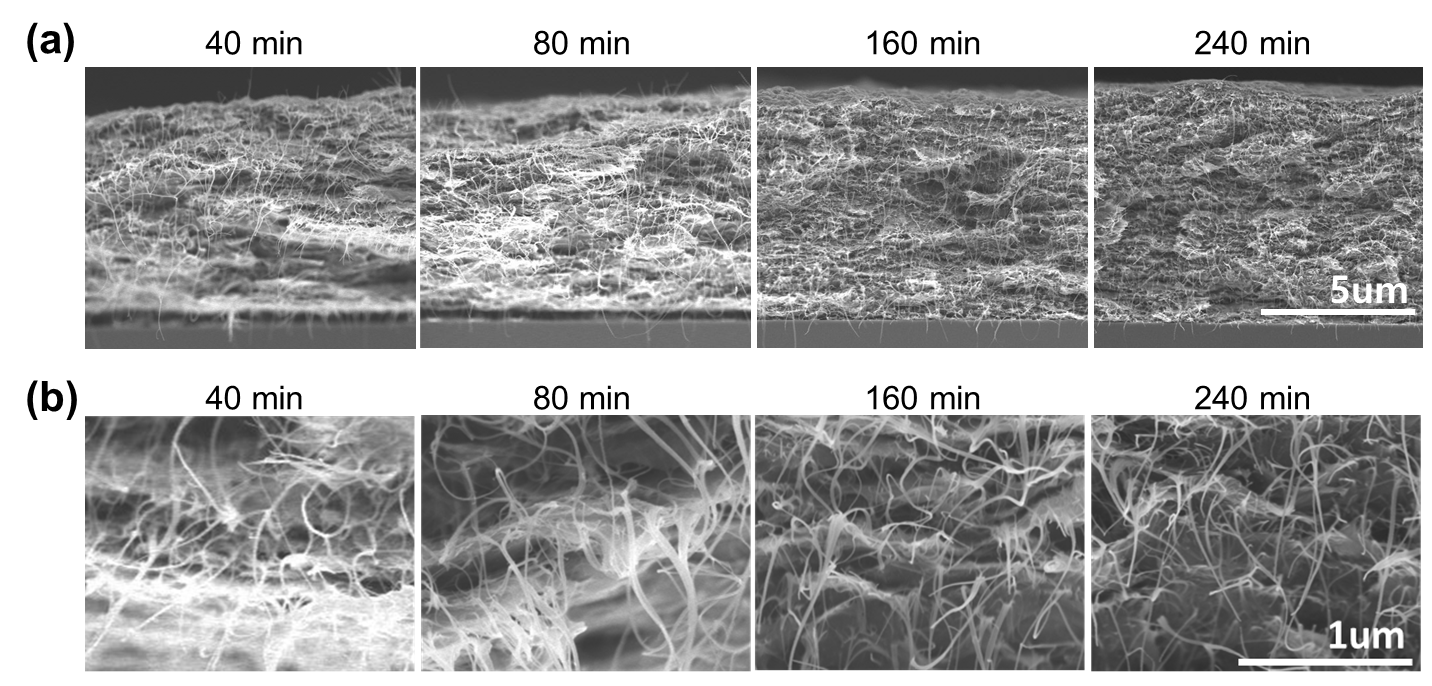
**

**Figure S5.** (a) The representative cross-sectional SEM images of C-CNT films with various shaking times for the thickness measurement. (b) The enlarged cross-sectional SEM images of C-CNT films depending on the shaking time.

**
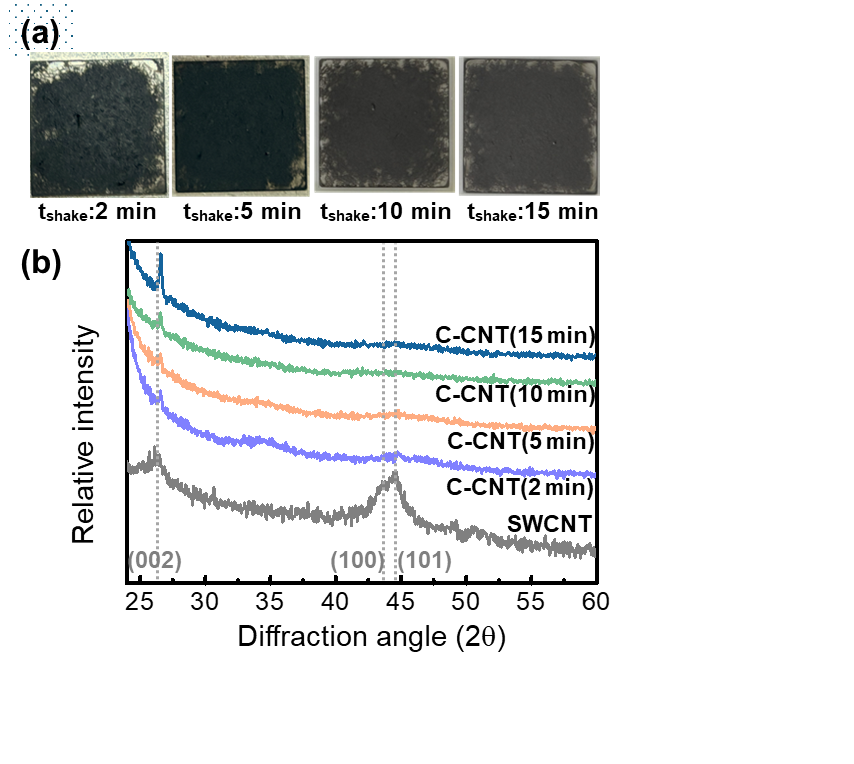
**

**Figure S6.** (a) Photograph of the drop-casted films using the dispersed C-CNT inks as increasing the shaking time from 2 min to 15 min. (b) X-ray diffraction (XRD) patterns of the SWCNTs and the C-CNT films obtained under various shaking times.

**
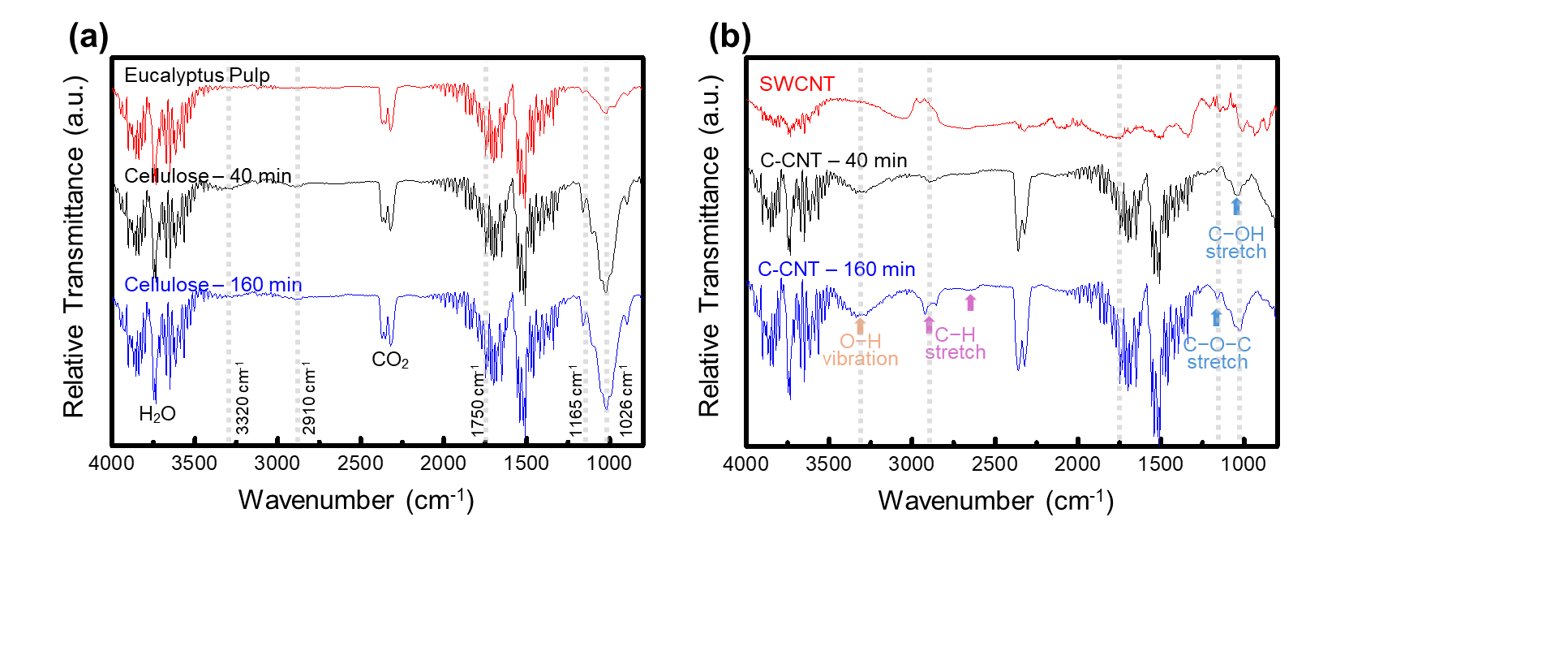
**

**Figure S7.** (a) FT-IR spectra of pristine Eucalyptus pulp and bare cellulose films obtained from the pulp with various shaking times. (b) FT-IR spectra of bare single walled carbon nanotubes (SWCNTs) and cellulose-SWCNT (C-CNT) films with various shaking times.

**
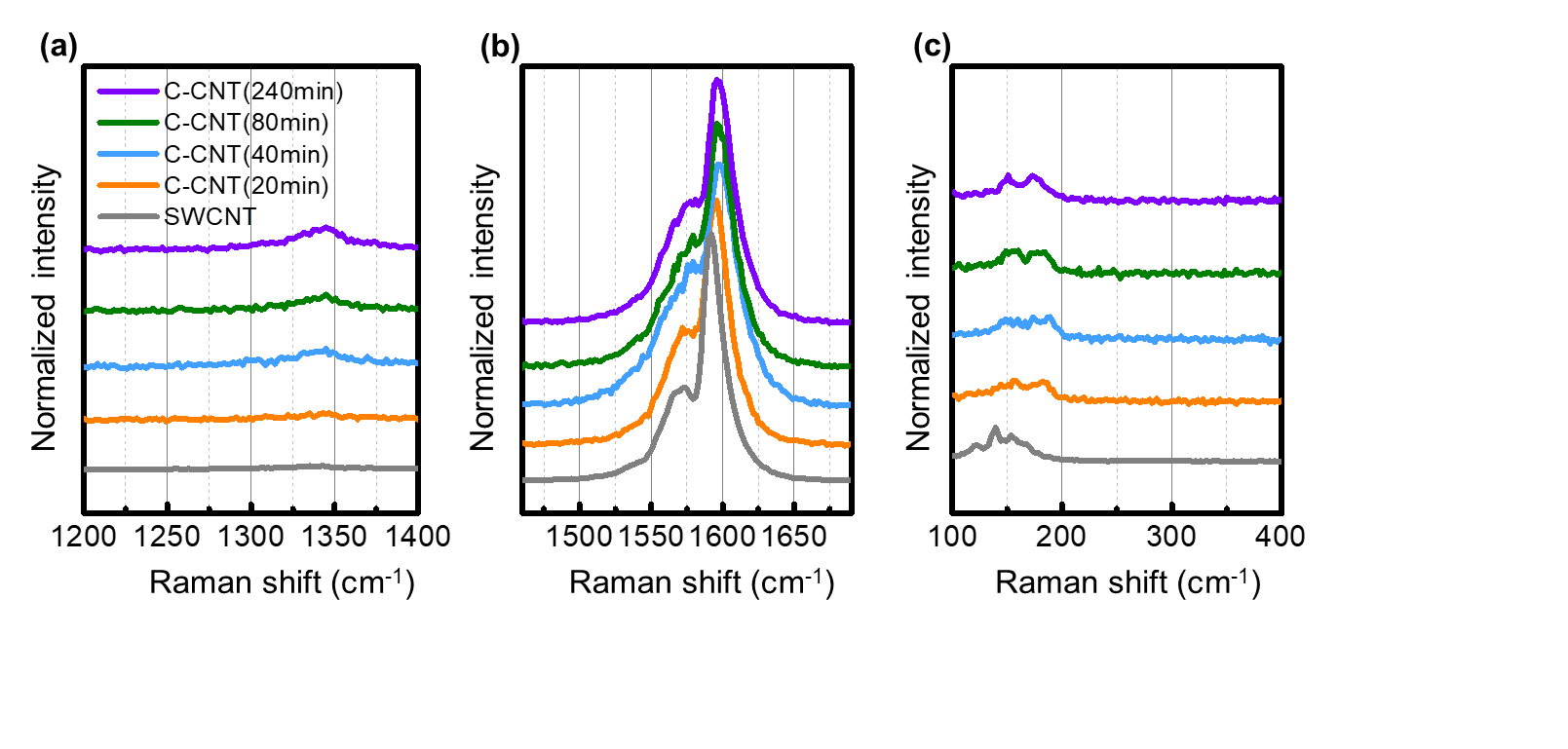
**

**Figure S8.** The enlarged Raman spectra of the tested samples in Figure 1d at (a) D-band peaks, (b) G-band peaks, and (c) radial-breathing-mode (RBM) peaks.

**
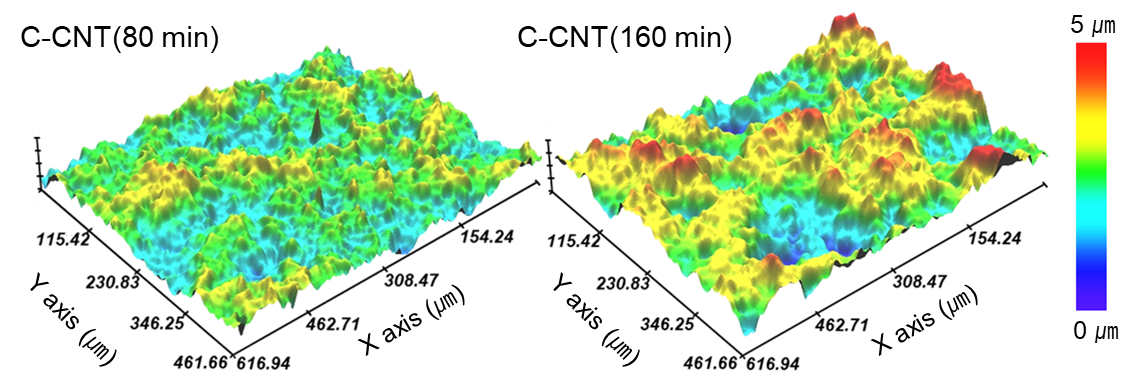
**

**Figure S9.** 3D surface morphological images of C-CNT films with the shaking time of 80, 160 minutes by using 3D optical surface profiler.


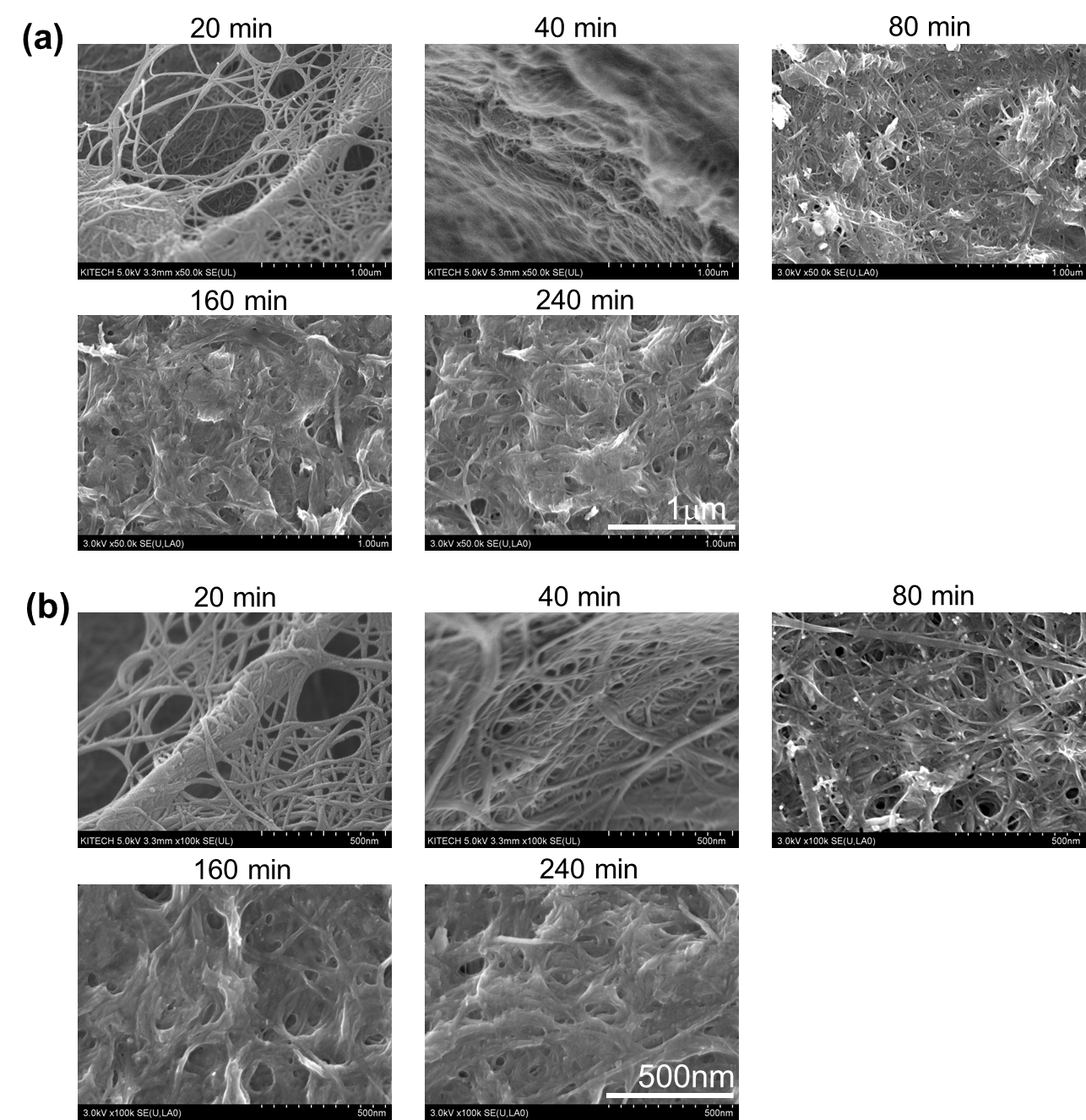


**Figure S10.** SEM images of C-CNT films with various shaking times using the magnification of (a) × 50,000 and (b) × 100,000.


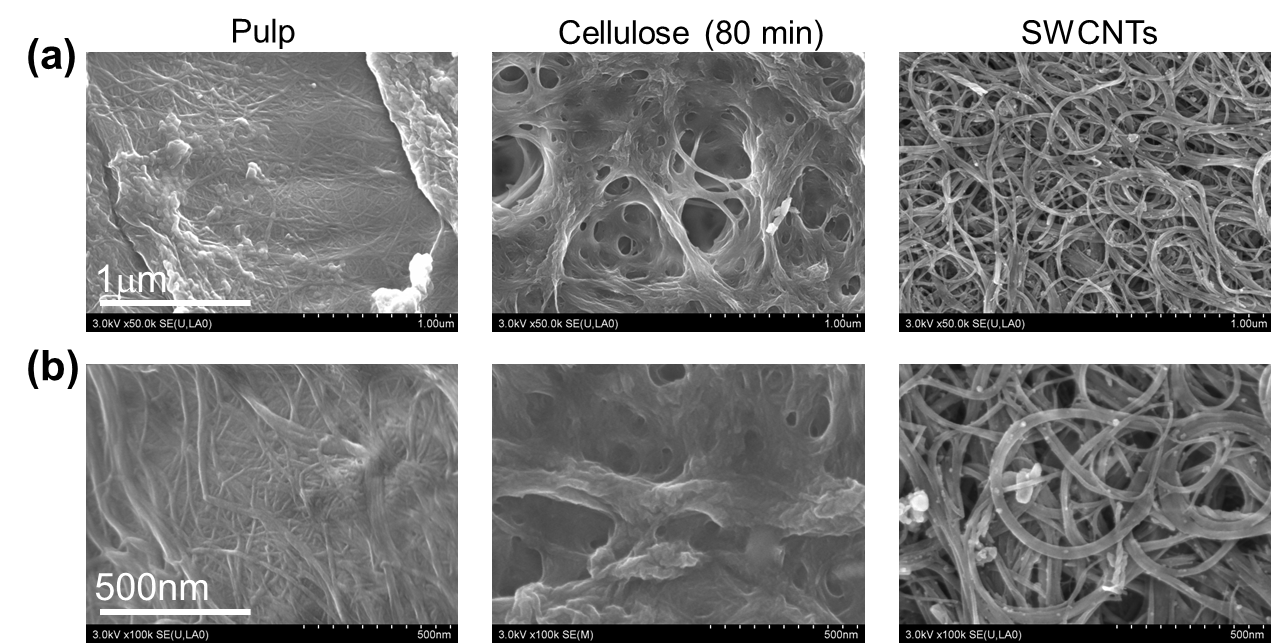


**Figure S11.** SEM images of the natural pulp, the cellulose film obtained by shaking the pulp for 80 minutes, and the single walled carbon nanotubes using the magnification of (a) × 50,000 and (b) × 100,000.


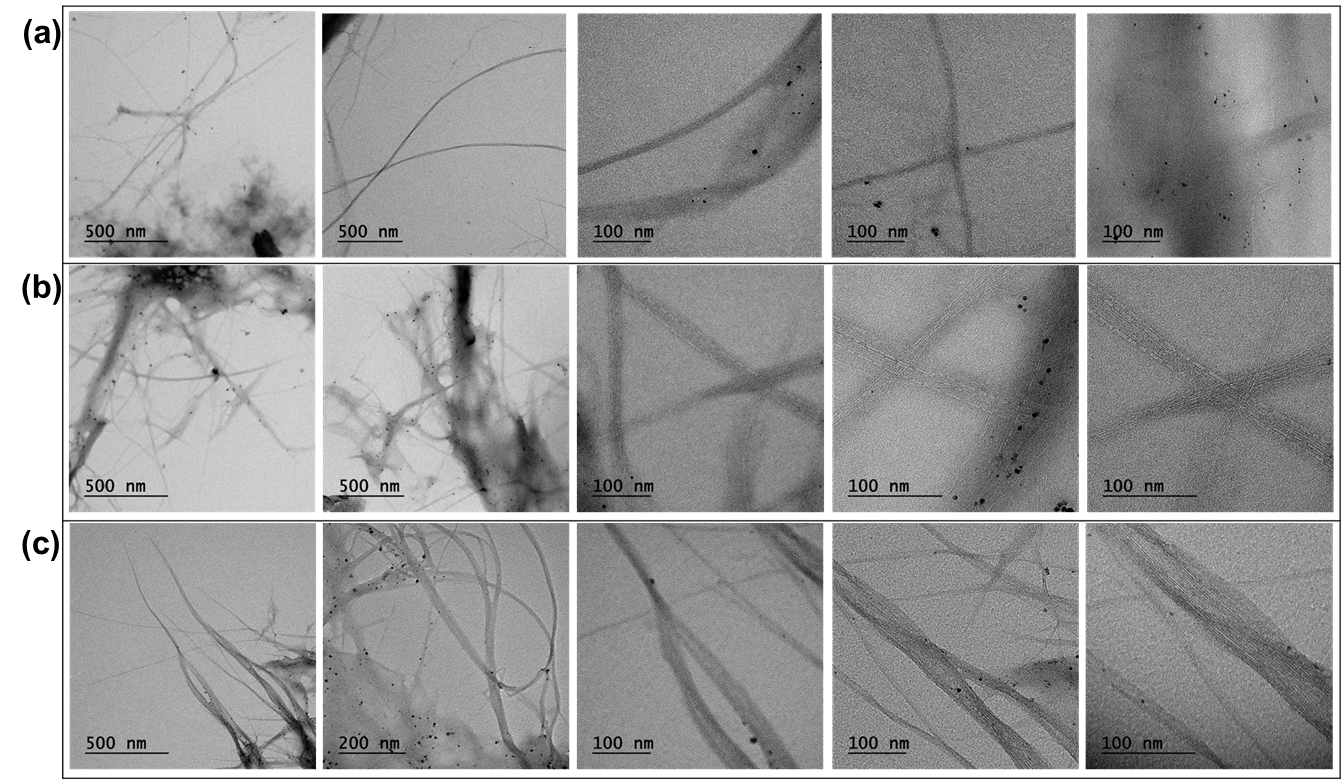


**Figure S12.** TEM images of the developed nanowires in the C-CNT film after shaking (a) 40 minutes, (b) 80 minutes, (c) 160 minutes with various magnifications. The nanowire-structures with the length over few micrometers can be found over the entire measuring region, where the developed C-CNT nanowires maintained the alternating structure based on SWCNT chain (the white line) and the nanocellulose chain (the black line).


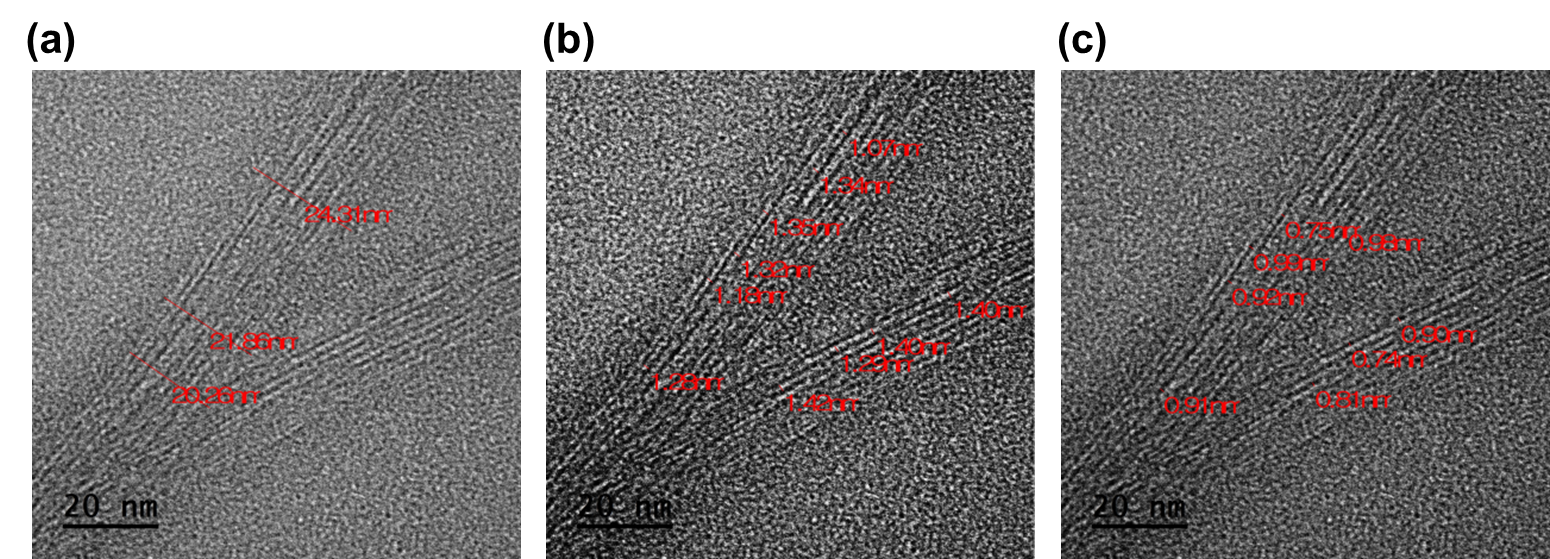


**Figure S13.** High resolution TEM images of the developed nanowires in the C-CNT film after shaking 40 minutes for measuring (a) the size of a single wire, (b) the size of alternating SWCNT chain (the white line), and (c) the size of alternating nanocellulose chain (the black line)


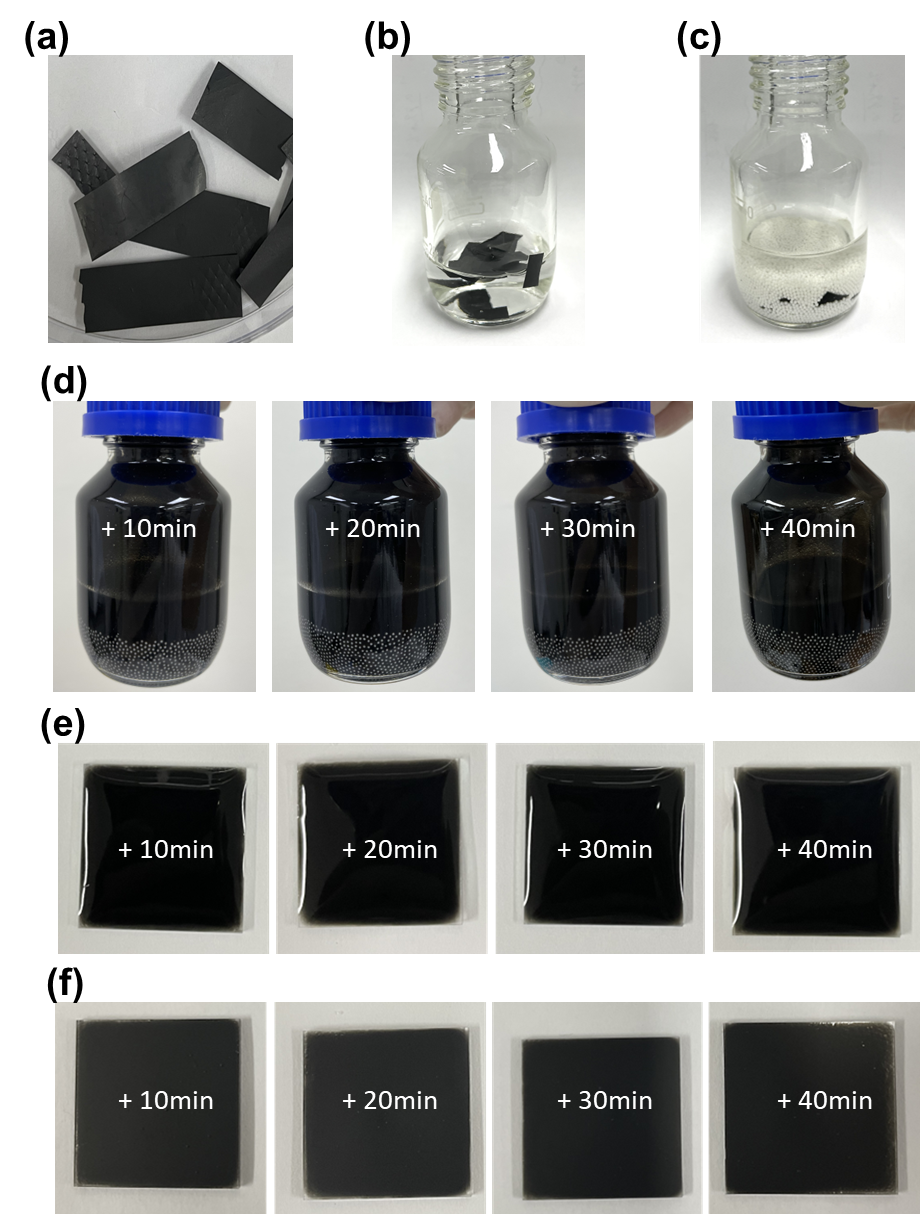


**Figure S14.** The reusable test for the completely dried C-CNT film according to the shaking time of 40 minutes. Photograph of aqueous suspensions of (a) the prepared C-CNT film with an electrical conductivity of 102 S/cm after testing mechanical properties. (b) the soaked and stirred C-CNT film for a week in water. (c) the C-CNT film with zirconia beads in water subjected to an ultrasonic homogenizer for 30 minutes. (d) Photograph of the re-dispersed C-CNT inks as increasing the additional shaking time from 10 minutes to 40 minutes. Photograph of the drop-casted films using the re-dispersed C-CNT inks. Pictures are taken (e) just after drop-casting 0.5 ml C-CNT ink on a 20 mm×20 mm glass substrate and (f) after completely drying the drop-casted film.
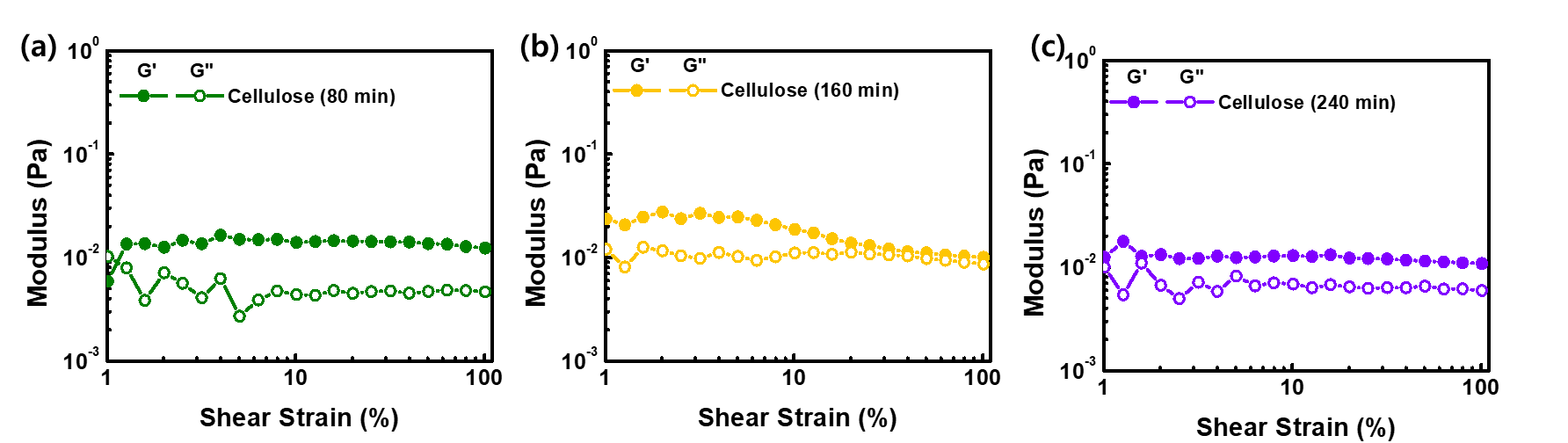


**Figure S15.** Storage moduli (*G’*) and loss moduli (*G”*) as a function of shaking time (a) 80 min, (b) 160 min, and (c) 240 min for cellulose 1 wt% solutions. (Open symbols are *G’* and solid symbols are *G”*.)


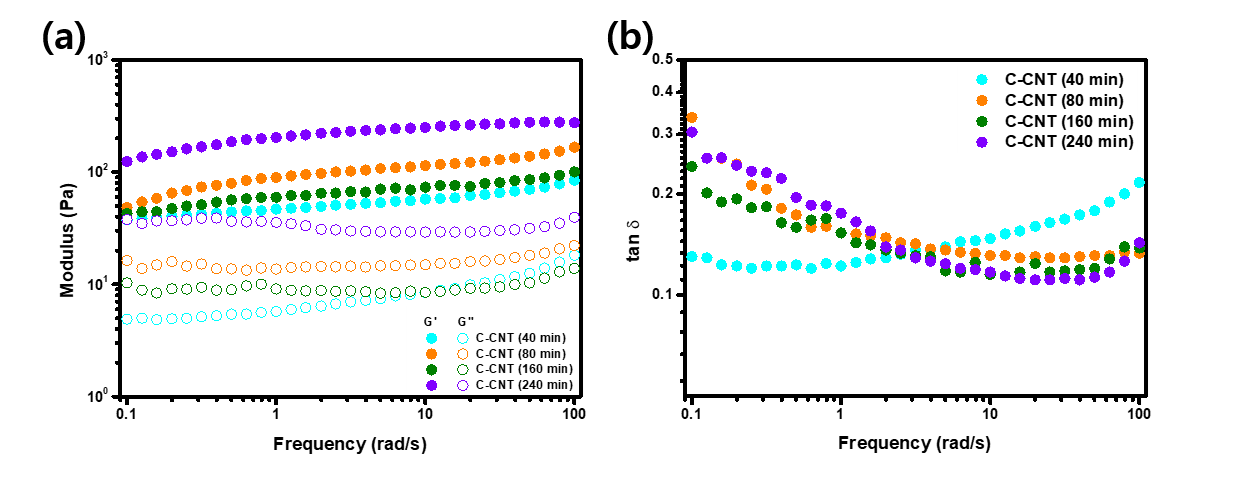


**Figure S16.** (a) Modulus (solid symbols are G' and open symbols are G"), and (b) tan δ (G"/G') plots obtained via frequency sweep test as a function of shaking time.


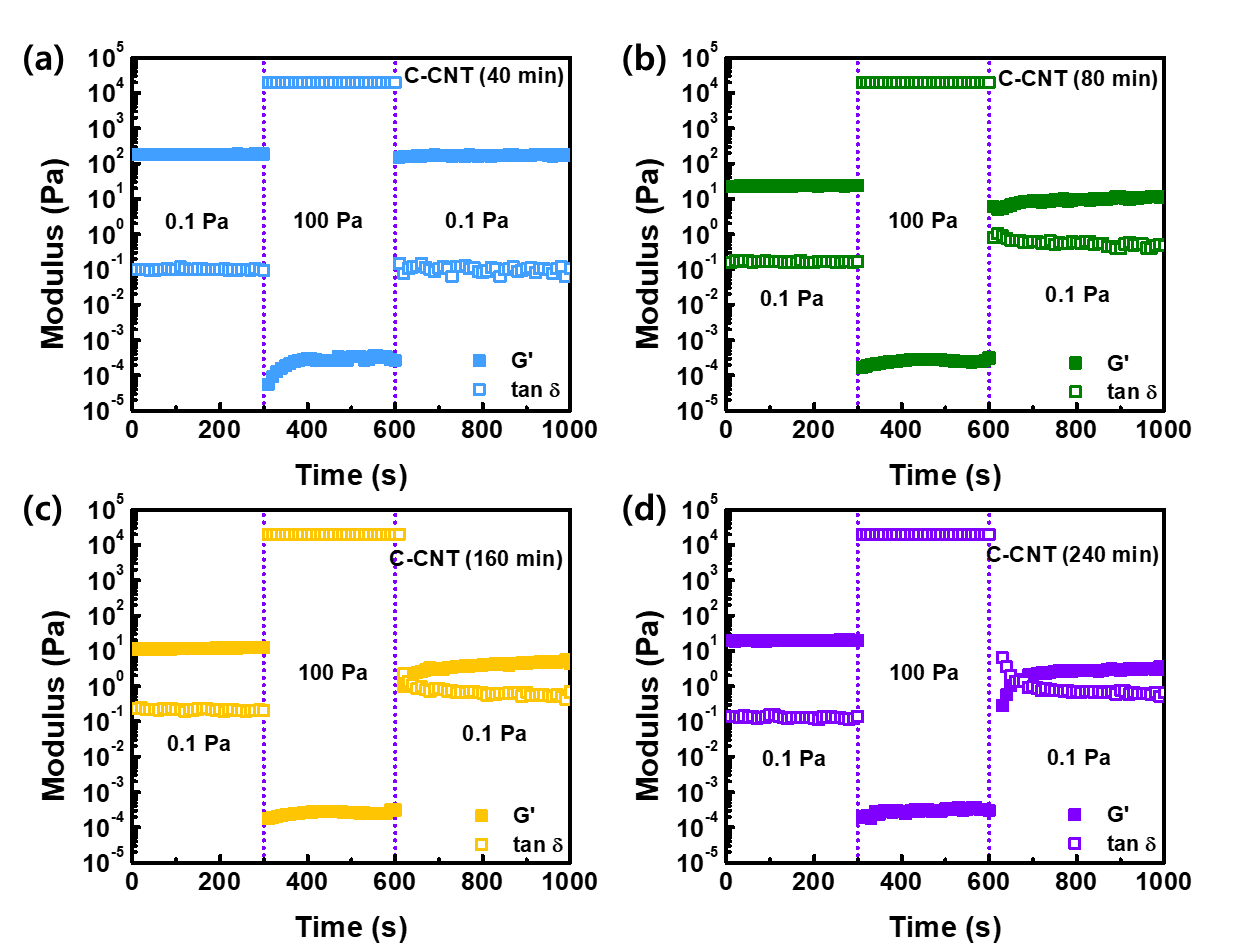


**Figure S17.** Three interval thixotropic tests (3ITT) for evaluating the coating property of C-CNT dispersions according to the shaking time (a) 40 min, (b) 80 min, (c) 160 min, and (d) 240 min.


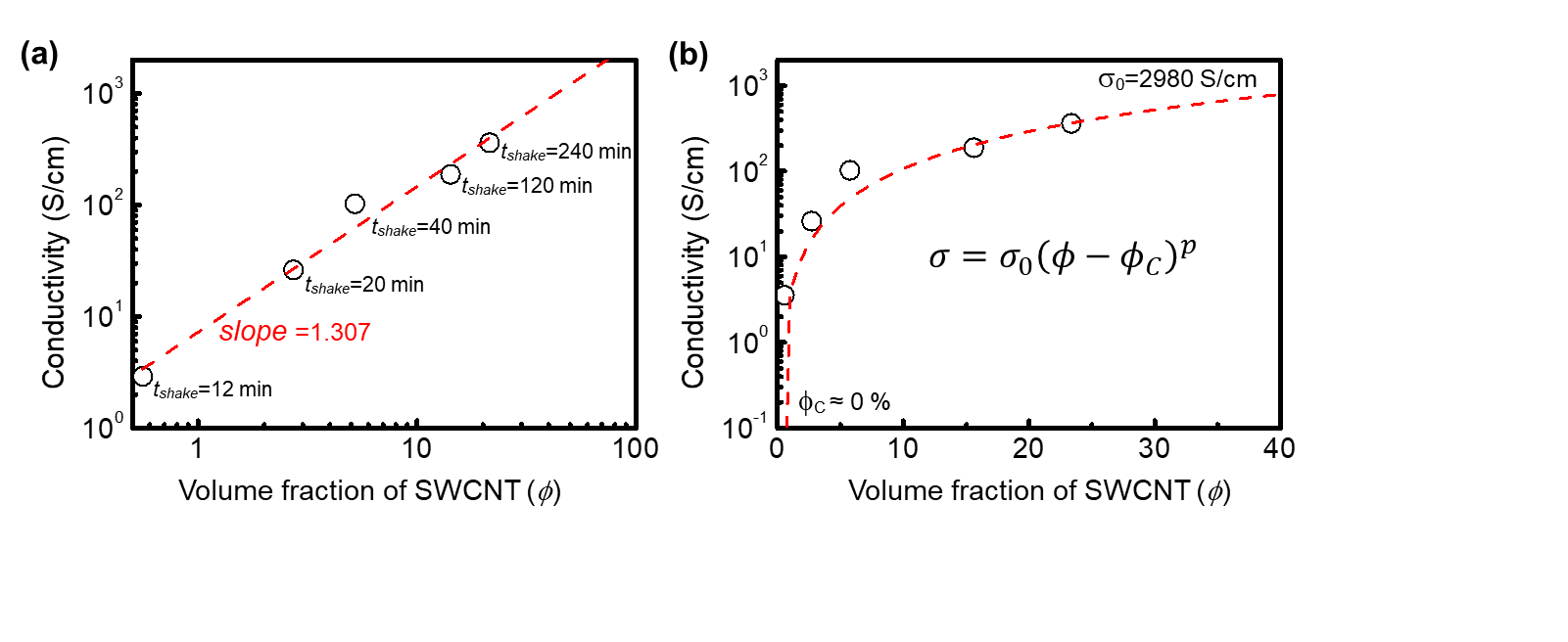


**Figure S18.** Plot of the measured maximum conductivity (S_max_) of C-CNT films as a function of volume fraction of SWCNTs (φ_SWCNT_) (a) log (S_max_) vs. log (φ_SWCNT_). Solid line corresponds to a linear fit to the experimental data. Dash lines means the fitted lines for selected data of lower φ_SWCNT_ (black) and higher φ_SWCNT_ (blue), respectively. (b) log (S_max_) vs. φ_SWCNT_. Solid line corresponds to a power law fit to the experimental data. The volume fraction of SWCNTs was calculated from weight using a density of 1.35 g cm^-3^ and 0.75 g cm^-3^ for SWCNT and bleached eucalyptus kraft pulp, respectively. The inset equation in (b) shows the relationship between conductivity (σ) and the volume fraction of SWCNT (φ) in power law percolation theory, where σ_0_ is the conductivity of the pure SWCNT, φ_C_ is the percolation threshold of the volume fraction and p is the critical exponent.


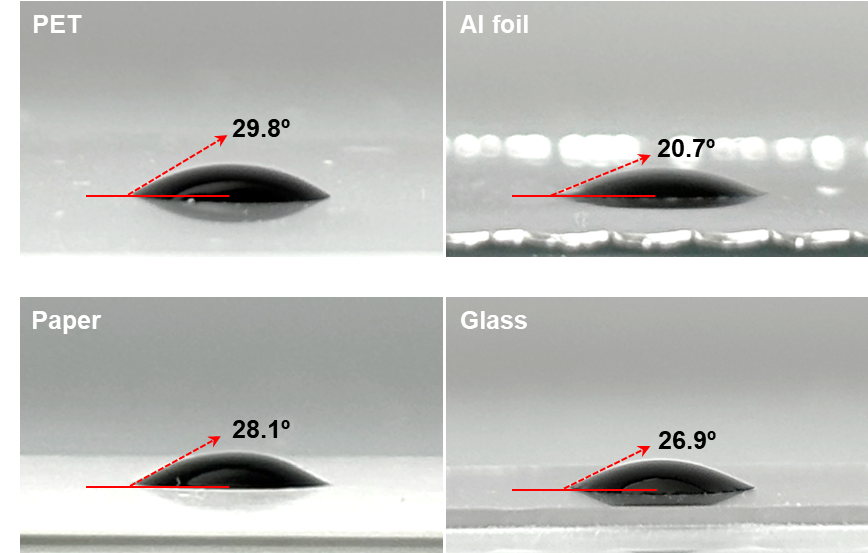


**Figure S19.** The pictures of the contact angle measurement on the diverse substrates. The contact angle was measured after dropping 20 μl of C-CNT ink with t_shake_ of 40 minutes.


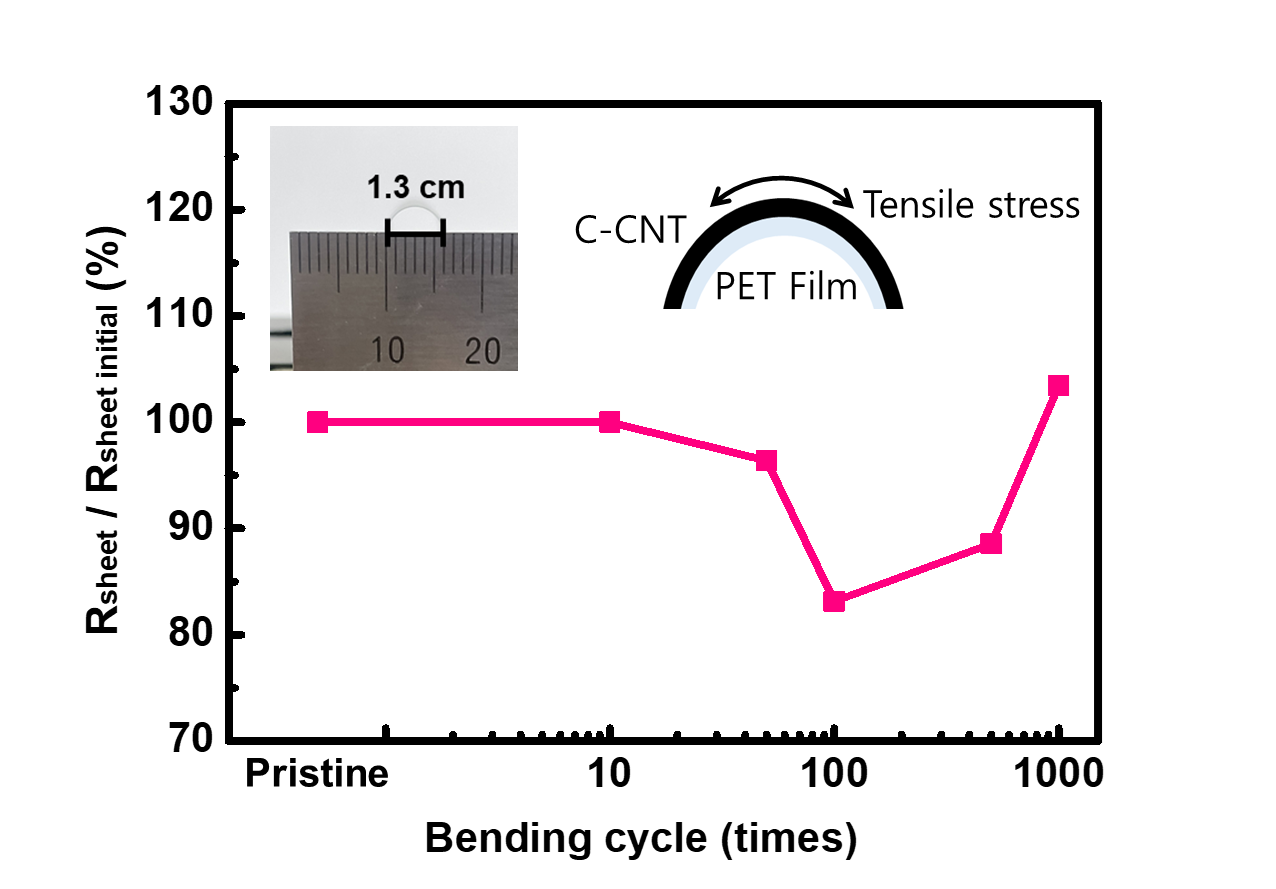


**Figure S20.** Sheet resistance value changes of the coated C-CNT film on a PET as a transparent electrode after each cycle with the bending radius of 6.5 mm.


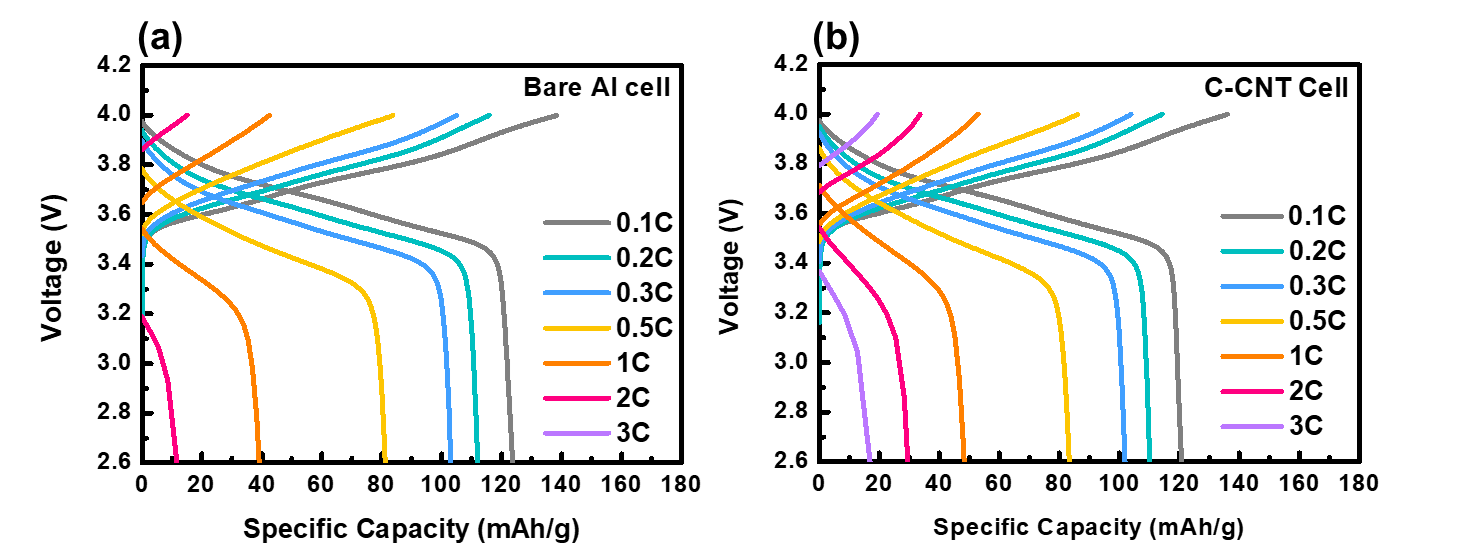


**Figure S21.** Charge/discharge curves of the tested all-solid-state batteries (ASSB) at 70 ℃ based on (a) the bare aluminum foil and (b) the 2 μm-thick C-CNT layer coated on aluminum foil as the cathode current collector. The charge–discharge measurements of the prepared 2032 coin-type cell were performed in the potential range from 2.5 to 4.0 V vs. Li^0^/Li^+^ as increasing current densities from 0.1C to 3C.


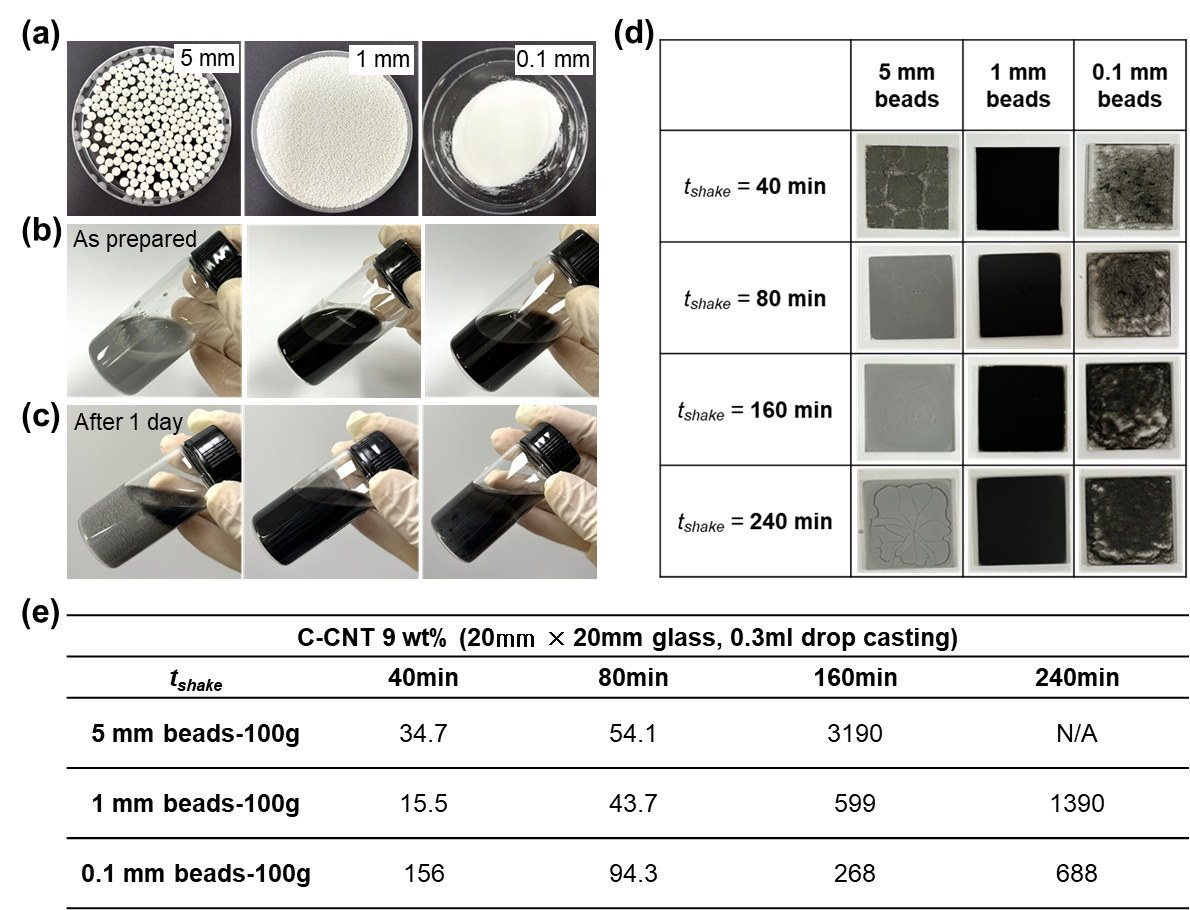


**Figure S22.** The influence of the size of zirconia beads on the preparation of C-CNT inks. (a) The tested size of zirconia beads with the diameter of 5 mm, 1 mm, and 0.1 mm. (b) Photograph of aqueous suspensions of C-CNT in de-ionized water after shaking for 40 minutes. (c) Photograph of aqueous C-CNT inks kept for 1 day after preparation. In the C-CNT ink with 5mm-diameter zirconia beads, the separation of cellulose and SWCNTs was observed. (d) The pictures of tested C-CNT films prepared by drop casting 0.3 ml of C-CNT ink on a glass substrate. C-CNT films from 0.1mm-diameter zirconia beads showed the poor coating uniformity even after shaking for 240 minutes. (e) The measured sheet resistance values for the tested C-CNT films after completely dried.


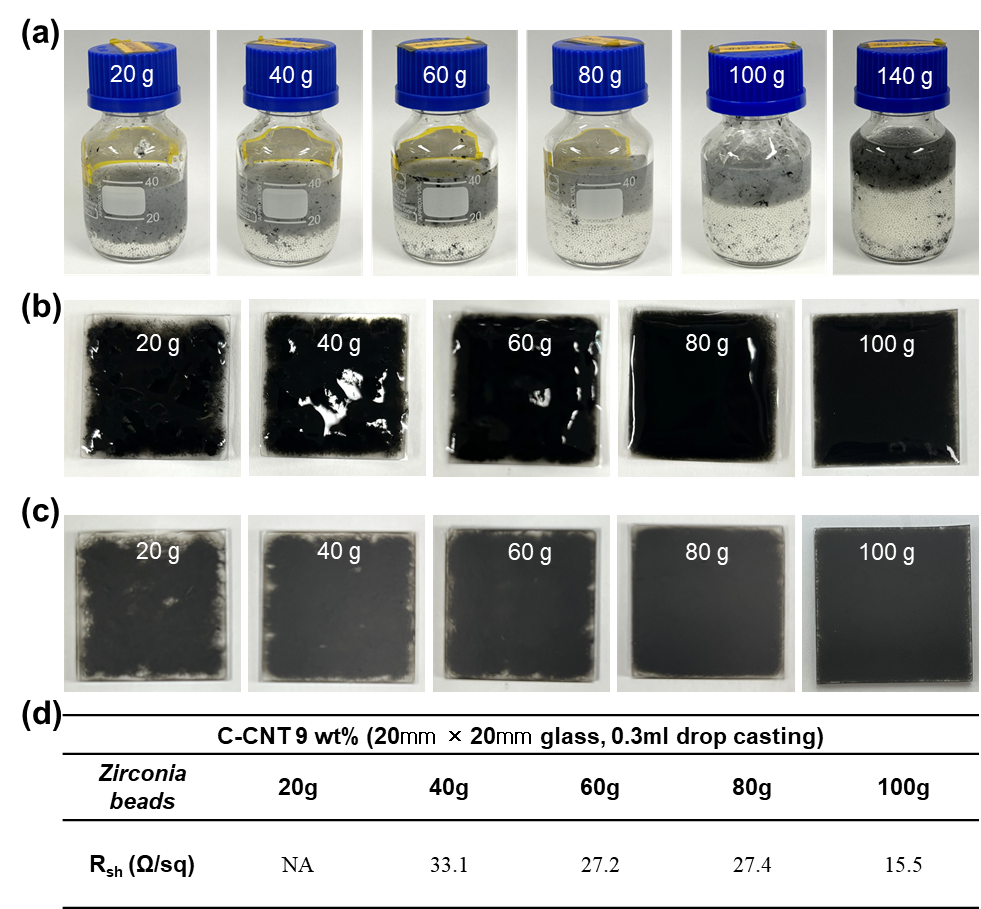


**Figure S23.** The influence of the amount of zirconia beads on the preparation of C-CNT inks. (a) Photograph of the mixture of the swollen pulp (0.3 g), SWCNT powder (0.03 g), and de-ionized water (30 ml) in the wide-neck glass bottle (50 ml) before the shaking process. We added various amounts of 1mm-zirconia beads onto the glass bottle. However, the total volume of the mixture for the batch with 140 g of zirconia beads exceeded the operation level of the used glass bottle. The shaking process was performed for 40 minutes. (b) The pictures of tested C-CNT inks after drop casting 0.3 ml of C-CNT ink onto a glass substrate. (c) The pictures of tested C-CNT films after completely dried. (d) The measured sheet resistance values for the dried C-CNT films.

**Table S1.** The measured zeta (ζ)-potentials of an aqueous solution based on SWCNTs only and C-CNTs were measured using electrophoretic light scattering method.

| **ζ-potentials for the various samples after shaking for 40 minutes** | | |
| --- | --- | --- |
| **Tested sample** | Only SWCNTs | C-CNTs |
| **Shaking time** | 40 min | 40 min |
| **ζ-Potential (mV)** | -3.59 | -25.31 |

**Table S2.** Apparent hydrodynamic diameter of C-CNT in an aqueous solution from the measured dynamic light scattering (DLS) data.

| **Hydrodynamic diameter of C-CNT aqueous solutions** | | | |
| --- | --- | --- | --- |
| **Shaking time** | 40 min | 80 min | 160 min |
| **Hydrodynamic diameter (nm)** | 1162 | 1609 | 2925 |

**Table S3.** The electrical characteristics of C-CNT films with various shaking times. The sheet resistance values were measured using Van der Pauws method for over 5 samples. And the film thickness was measured from the cross-sectional SEM images over 6 points. The thickness for 20-, 60-, 120-, and 200-minutes samples was estimated using nearest measurement results. The electrical conductivity was obtained using average value of sheet resistance and film thickness.

| **The drop-casted C-CNT film with 9 wt% SWCNTs** | | | | | | | | |
| --- | --- | --- | --- | --- | --- | --- | --- | --- |
| **Shaking time** | 20min | 40min | 60min | 80min | 120min | 160min | 200min | 240min |
| **Sheet resistance**  **(Ω/sq)** | 17.8  (±1.77) | 15.5  (±1.20) | 26.5  (±1.05) | 43.7  (±0.13) | 84.9  (±0.22) | 599  (±0.36) | 883  (±8.7) | 1390  (±2.84) |
| **Thickness**  **(μm)** | 6.3 | 6.3  (±0.20) | 6.5 | 6.7  (±0.15) | 6.9 | 7.4  (±0.13) | 7.7 | 7.9  (±0.10) |
| **Conductivity (S/cm)** | 91.8 | 102.4 | 59.9 | 34.2 | 17.1 | 2.6 | 1.5 | 0.9 |

**Table S4.** The sheet resistance values of re-dispersed C-CNT films with various additional shaking times (Figure S11). The sheet resistance values were measured using Van der Pauws method for over 5 samples. The data for the shaking time of 40 min(pristine), 60 min, and 80 min came from Table S1 for the comparison.

| **The drop-casted C-CNT film with 9 wt% SWCNTs** | | | | | | | |
| --- | --- | --- | --- | --- | --- | --- | --- |
| **Shaking time** | 40min **(pristine)** | +10min **(reuse)** | +20min **(reuse)** | +30min **(reuse)** | +40min **(reuse)** | 60min **(pristine)** | 80min **(pristine)** |
| **Total shaking time** | 40min | 50min | 60min | 70min | 80min | 60min | 80min |
| **Sheet resistance**  **(Ω/sq)** | 15.5 (±1.20) | 22.4 (±0.20) | 24.8 (±0.09) | 33.7 (±0.23) | 44.0 (±0.23) | 26.5 (±1.05) | 43.7 (±0.13) |
| **Conductivity (S/cm)** | 102.4 | 70.8 | 64.0 | 45.7 | 33.9 | 59.9 | 34.2 |

**Table S5.** The values for the discharge capacity and the corresponding discharge capacity retention, which were obtained from the charge/discharge test of the prepared all-solid-state batteries (ASSB) in Figure 5c depending on the current density.

|  | **ASSB with C−CNT film coated on Al foil** | | **ASSB with bare Al foil** | |
| --- | --- | --- | --- | --- |
| **Current density (C-rate)** | Discharge Cap. (mAh/g) | Discharge Cap. retention. (%) | Discharge Cap. (mAh/g) | Discharge Cap. retention. (%) |
| **0.1 C** | 121.19 | 100 | 124.1 | 100 |
| **0.2 C** | 110.44 | 91.1 | 112.3 | 90.5 |
| **0.3 C** | 102.15 | 84.3 | 103.3 | 83.3 |
| **0.5 C** | 83.63 | 69.0 | 81.44 | 65.6 |
| **1.0 C** | 48.69 | 40.2 | 39.50 | 31.8 |
| **2.0 C** | 30.04 | 24.8 | 12.03 | 9.7 |
| **3.0 C** | 17.45 | 14.4 | NA | NA |
| **0.1 C** | 121.37 | 100 | 123.21 | 99.3 |
